# Calycosin Targets the CYP1B1‐AKT/SP1‐GPX4 Axis to Modulate Ferroptosis in Colorectal Carcinogenesis

**DOI:** 10.1002/ptr.70172

**Published:** 2026-01-06

**Authors:** Lihan Bie, Xin Lei, Di Wu, Yang Zhang, Chengshan He, Luyao Liu, Jiawei Zhou, Xin Zhou, Yingying Lu, Zheng Xu

**Affiliations:** ^1^ Department of Clinical Laboratory Seventh People's Hospital of Shanghai University of Traditional Chinese Medicine Shanghai People's Republic of China; ^2^ Department of Clinical Laboratory Yangpu Hospital, School of Medicine, Tongji University Shanghai People's Republic of China; ^3^ School of Medicine, Anhui University of Science and Technology Huainan Anhui People's Republic of China

**Keywords:** Calycosin, CRC, CYP1B1, ferroptosis, GPX4

## Abstract

Calycosin, a natural flavonoid small‐molecule compound derived from traditional Chinese medicine, has demonstrated remarkable pharmacological activity in the field of cancer therapy. This study systematically elucidates the molecular mechanisms of Calycosin in colorectal cancer (CRC) treatment through integrated in vivo and in vitro experiments. In vivo experiments revealed that Calycosin effectively inhibits subcutaneous tumor growth in CRC‐bearing mice. In vitro assays and transcriptome sequencing confirmed that Calycosin effectively suppresses migration, invasion, epithelial‐mesenchymal transition (EMT), and induces ferroptosis in human CRC cells, thereby inhibiting malignant tumor behaviors. Cellular Thermal Shift Assay (CETSA) and site‐directed mutagenesis experiments first identified cytochrome P450 1B1 (CYP1B1) and Gly‐329 as critical binding targets and sites for Calycosin. Functional studies showed that CYP1B1 knockdown in vitro and in vivo suppresses GPX4 expression and enhances ferroptosis in CRC cells. Mechanistically, CYP1B1 activates the AKT/SP‐1 signaling pathway to upregulate GPX4 expression, thereby modulating colorectal carcinogenesis and progression. In summary, this study first unveils the crucial role of Calycosin and the CYP1B1‐AKT/SP1‐GPX4 regulatory axis in CRC ferroptosis, providing novel theoretical foundations for targeted therapy using traditional Chinese medicine‐derived small molecules against colorectal cancer.

## Graphical abstract

Mechanism of Calycosin in Regulating Ferroptosis in Colorectal Cancer via Binding to CYP1B1.

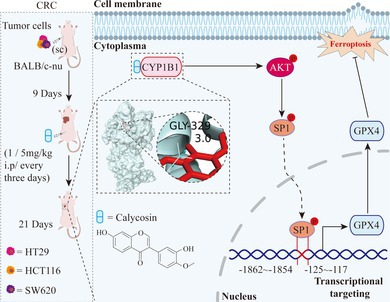

**Abbreviations**
AKR1C1: aldo‐keto reductase family 1 member C1
CETSA: cellular thermal shift assay
CRC: colorectal cancer
CYP1B1: cytochrome P450 1B1
ECC: extrahepatic cholangiocarcinoma
EMT: epithelial‐mesenchymal transition
FTH1: ferritin heavy chain 1
GPX4: glutathione peroxidase 4
OS: overall survival
SP1: specificity protein 1
TCM: Traditional Chinese medicine
TNBC: triple‐negative breast cancer

## 1 Introduction

Colorectal Cancer (CRC), as the third most common malignancy globally, has an annual incidence of over 1.9 million new cases, with a 5‐year overall survival rate of only approximately 57% (Abedizadeh et al. 2024). Based on anatomical characteristics, CRC can be categorized into colon cancer (accounting for 70%) and rectal cancer (30%). These two subtypes exhibit significant heterogeneity in terms of molecular classification, metastasis patterns, and treatment sensitivity (Li et al. 2024). The current clinical standard treatment primarily consists of radical surgical resection, combined with neoadjuvant chemoradiotherapy and pharmacological therapy (Zhu and Benson 3rd 2024). Among pharmacological treatments, traditional chemotherapy drugs such as 5‐FU/LV (with an anemia incidence of 15%–30%) and oxaliplatin (with a peripheral neurotoxicity incidence of ≥ 50%) often cause significant toxic and side effects, severely impacting patients' quality of life (Jin et al. 2024). Although immune checkpoint inhibitors (PD‐1/PD‐L1 inhibitors) have shown groundbreaking efficacy in dMMR/MSI‐H type CRC (with an ORR reaching 40%), approximately 85% of pMMR/MSS patients still exhibit primary resistance (Duta‐Ion et al. 2024).

Small‐molecule compounds from traditional Chinese medicine (TCM) demonstrate distinct advantages in anticancer drug development due to their multi‐target intervention strategies and inherent low toxicity profiles (Zou et al. 2024). Chemically classified, these compounds primarily encompass over 10 categories including alkaloids, flavonoids, and saponins (Jin et al. 2025). Flavonoids and saponins have emerged as focal points in current antitumor research due to their straightforward purification processes, well‐defined chemical structures, and cost‐effective production (Galati and O'Brien 2004). This study focuses on three representative TCM small molecules: (1) (+)‐Gallocatechin, a flavonoid from green tea C, which inhibits tumor growth via anti‐angiogenic and antioxidant effects through key enzyme modulation (Alam et al. 2022; Hu et al. 2024); (2) Calycosin, an isoflavonoid from Leguminosae plants, induces tumor cell apoptosis through multi‐pathway regulation involving ERβ/MiR‐95 and IGF‐1R/PI3K/Akt signaling pathways (Li, Lu, et al. 2023; Li, Hu, et al. 2023); (3) Ziyuglycoside I, a saponin compound, mediates p53‐dependent cell cycle arrest and activates caspase cascade to enhance pro‐apoptotic Bax expression in triple‐negative breast cancer (TNBC) (Zhu et al. 2018, 2016). Furthermore, SH‐PEG‐NH2‐coated nanoparticles loaded with Ziyuglycoside I have been shown to promote autophagy in hematopoietic stem cells, offering therapeutic potential for tumor patients with bone marrow suppression (Xiong et al. 2023).

To elucidate the anti‐CRC mechanisms of (+)‐Gallocatechin, Calycosin, and Ziyuglycoside I, we established subcutaneous xenograft models using three human CRC cell lines (HT29, HCT116, SW620), identifying Calycosin as the optimal candidate. Subsequent in vitro models integrated with transcriptomic sequencing systematically analyzed Calycosin's effects on CRC cellular phenotypes and functionalities. Target prediction databases, molecular docking, Cellular Thermal Shift Assay (CETSA), and site‐directed mutagenesis were employed to identify Calycosin's binding targets and interaction sites. Finally, in vitro knockdown/overexpression models and in vivo target‐silenced animal models were utilized to delineate the mechanistic roles of identified targets. This systematic approach provides robust evidence for understanding Calycosin's therapeutic functions and mechanisms in CRC, facilitating novel treatment development.

## 2 Materials and Methods

### 2.1 Main Chemicals and Reagents

(+)‐Gallocatechin (Cat. HY‐N0521A), Ziyuglycoside I (#HY‐N0331), and Calycosin (#HY‐N0519) were synthesized by MCE Company. Considering the safety and efficacy of the drugs, the dosage used in in vivo experiments was 5 mg/kg. For in vivo experiments, the solution was prepared with 10% DMSO +90% (20% SBE‐β‐CD). SBE‐β‐CD (#HY‐17031) was synthesized by MCE Company and was prepared into a 20% concentration with saline solution before use. In in vitro cell experiments, Calycosin was prepared into a 5 mM stock solution using DMSO (MCE, # HY‐Y0320), and the working concentration was 100 μM. The ferroptosis inhibitor Ferrostatin‐1 (Cat. HY‐100579) was synthesized by MCE Company, and the concentration for cell experiments was 10 μM. The AKT kinase inhibitor MK‐2206 2HCl (Cat. S1078) was synthesized by Selleck Company, and the concentrations for experiments on HT29 and SW620 cells were 66 and 200 μM, respectively.

### 2.2 Cell Line

Human colorectal cancer cells HT29 (#CL‐0118) and HEK‐293 (#CL‐0001) were purchased from Pricella Company. Human colorectal cancer cells HCT116 (#TCHu 99) and SW620 (#TCHu101) were purchased from the Cell Bank of the Chinese Academy of Sciences. HT29, HCT116, and SW620 cells were cultured in high‐glucose DMEM (Gibco, #C11995500BT) complete medium containing 10% FBS and 100 U/mL Penicillin/Streptomycin (P/S). HEK‐293 cells were cultured in MEM (Pricella, #PM150467) medium with 10% FBS and 100 U/mL P/S. All cells were cultured at 37°C in a constant temperature and humidity environment with 5% CO_2_.

### 2.3 Construction of Subcutaneous Animal Models for Colorectal Cancer

A total of 110 specific pathogen‐free (SPF) BALB/c‐nu mice (6–8 weeks old; 18–20 g) were purchased from Henan Skbiosa Biotechnology Co. Ltd. (SCBS). All mice were housed in the Experimental Animal Center of the Seventh People's Hospital of Shanghai, maintained at a temperature of 20°C ± 3°C, humidity of 55% ± 5%, and a 12‐h light/dark cycle, with daily observations and provision of food and drinking water. After acclimatizing for 7 days, the mice were randomly grouped for the experiment.

In terms of drug dosage selection, it is known that in normal rats, there are no significant toxic reactions to oral administration of (+)‐Gallocatechin at 50–200 mg/kg (Shi et al. 2022). In a sepsis rat model, intraperitoneal injection of 10 mg/kg of Ziyuglycoside I once daily for 7 days did not show apparent toxic reactions (Xiao et al. 2022). In pharmacokinetic studies, intravenous injection of 1 mg/kg of Ziyuglycoside I into normal rats exhibited no evident toxicity (Shen et al. 2024). In a mouse model, Calycosin was administered intraperitoneally at a dose range of 1–4 mg/kg once daily for 20 days without notable toxic reactions (Chen et al. 2022). In a rat model, oral administration of 7.5–30 mg/kg of Calycosin once daily for 3 days demonstrated neuroprotective effects against cerebral ischemia/reperfusion injury in adult male Sprague–Dawley rats (Xu et al. 2023). Based on bioavailability conversions for different administration routes (oral: intraperitoneal: intravenous = 1:0.35:0.25) and equivalent dose conversions between rats and mice using body surface area (BSA), the maximum safe doses of (+)‐Gallocatechin, Ziyuglycoside I, and Calycosin administered intraperitoneally to mice were calculated as 101.1, 14.4, and 15.1 mg/kg, respectively. Additionally, considering the effective dose, a final experimental dose of 5 mg/kg was chosen. Furthermore, this study has fully considered the relevant requirements outlined in recent guidelines for best practice in pharmacological research on natural products (Heinrich et al. 2020; Wang et al. 2024).

For the treatment of subcutaneous colorectal cancer mouse models with (+)‐Gallocatechin (5 mg/kg), Ziyuglycoside I (5 mg/kg), and Calycosin (5 mg/kg), 60 mice were divided into three drug treatment groups for HT29, HCT116, and SW620 models. Each model consisted of four groups: PBS and (+)‐Gallocatechin treated HT29/HCT116/SW620, Ziyuglycoside I treated HT29/HCT116/SW620, and Calycosin treated HT29/HCT116/SW620, with five mice in each group. In the PBS group, each mouse received a subcutaneous injection of 1 × 10^6^ HT29/HCT116/SW620 cells. On the 9th day after subcutaneous cell injection, the treatment groups received intraperitoneal injections of 5 mg/kg of the respective drugs (every 3 days).

In the toxicity evaluation model of (+)‐Gallocatechin, Ziyuglycoside I and Calycosin, mice were intraperitoneally injected for 18 days at a concentration of 5 mg/kg, with body weight recorded every 3 days. After the end of the model, the mice were anesthetized. Blood, heart, liver, spleen, lung, kidney, and brain tissues were collected. An automatic biochemical analyzer (BS‐240, Shenzhen Mindray Bio‐Medical Electronics Co. Ltd.) was used to determine the content of alanine aminotransferase (ALT) and aspartate aminotransferase (AST) in the blood. HE staining was used to detect the pathological changes of heart, spleen, lung, and kidney tissues. Oil red staining was used to detect the pathological changes of liver tissue, and Nissl staining was used to detect the pathological changes of brain tissue.

In the mouse model examining the effects of CYP1B1 knockdown on the growth of colorectal cancer in vivo, stable cell lines with CYP1B1 knockdown were established for HT29 (sh_CYP1B1_HT29) and SW620 (sh_CYP1B1_SW620), along with their respective controls (sh_NC). Thirty mice were divided into two mouse models to investigate the impact of CYP1B1 knockdown in HT29/SW620 cells on colorectal cancer growth in vivo. Each model comprised three groups: sh_NC_HT29/SW620, sh_CYP1B1_HT29/SW620, and sh_CYP1B1_HT29/SW620 + Calycosin, with five mice per group. The sh_NC_HT29/SW620 group received a subcutaneous injection of 1 × 10^6^ HT29/SW620 negative control (sh_NC) cells. The sh_CYP1B1_HT29/SW620 group was injected subcutaneously with 2 × 10^6^ CYP1B1‐knockdown HT29/SW620 cells. The sh_CYP1B1_HT29/SW620 + Calycosin group received a subcutaneous injection of 1 × 10^6^ CYP1B1‐knockdown HT29/SW620 cells, followed by an intraperitoneal injection of 5 mg/kg Calycosin on the 9th day (three times every 3 days). Tumor volumes were recorded every 3 days after tumor formation, calculated using the formula *V* = *π*/6 × *L*(Length) × *W*(Width)^2^. The model was terminated after 27 days, and the mice were euthanized by cervical dislocation under deep anesthesia with 1.5% isoflurane (RWD, #R510‐22‐10).

In this study's animal model, mice were euthanized immediately if they experienced a ≥ 20% weight loss, severe dyspnea, loss of mobility, or if the tumor volume exceeded 1200 m^3^ (no animals in this study met the criteria for early euthanasia). Additionally, the animal experiments were approved by the Medical Ethics Committee of the Seventh People's Hospital of Shanghai (Ethics Approval Number: 2025‐AR‐002).

### 2.4 Scratch Assay and Transwell Experiment

In the scratch assay, cells were digested with trypsin, centrifuged, resuspended, and counted. The cell concentration was adjusted to 3 × 10^5^ cells/mL, and they were seeded into 6‐well plates with three replicates per group. When cell confluence exceeded 90%, a parallel scratch was made in each well using a 10 μL pipette tip. Old media and floating cells were washed away with PBS, and serum‐free DMEM media was added. Scratch widths were observed and photographed under a microscope at 0 and 24 h. The scratch distances were measured using Image J (V1.8.0) software, and the cell migration rate (%) was calculated as (0 h width–24 h width)/0 h width × 100%.

In the Transwell experiment, cell invasion (with Matrigel) and migration (without Matrigel) were assayed. The upper chamber of the Transwell insert was pre‐coated with Matrigel (Corning Matrigel, #356234) diluted 1:9 in serum‐free media to simulate the in vivo extracellular matrix environment. Cell suspensions were prepared at a concentration of 1 × 10^5^ cells/mL, and 0.2 mL was seeded into the upper chamber of each insert. The lower chamber was filled with DMEM media containing 10% fetal bovine serum. After incubation for 24 h at 37°C and 5% CO_2_, the inserts were removed and washed with PBS to remove non‐migrated cells and media residues. Cells were then fixed with 4% paraformaldehyde for 15 min and stained with 0.1% crystal violet (Sigma‐Aldrich, #32675). Three random fields were selected under a microscope, and the number of cells in each field was recorded.

### 2.5 Flow Cytometry Detection of Apoptosis

Collect the treated human colorectal cancer cells and resuspend the cells in pre‐cooled PBS. Centrifuge at 300× *g* for 5 min, discard the supernatant, and collect the cells. Add 300 μL of 1× Binding Buffer to resuspend the cells and adjust the cell concentration to 1 × 10^6^/mL. Staining was performed using the Annexin V/PI kit (Key GEN, #KGA107). Take a flow tube, add 100 μL of filtered cell suspension, add 5 μL of Annexin V‐Alexa Fluor and gently mix; then add 10 μL of PI and gently mix again. Incubate at room temperature in the dark for 10 to 20 min, add 400 μL of 1× Binding Buffer, and detect with the machine. Data were processed using Flowjo.V10 software.

### 2.6 Transcriptome Sequencing and Analysis

Human colorectal cancer cells were collected after treatment, and RNA was isolated and purified using TRIzol reagent (Thermo Fisher, #15596018). The quantity and purity of total RNA were determined using a NanoDrop ND‐1000, while RNA integrity was assessed with a Bioanalyzer 2100. The required specifications for downstream experiments were a concentration > 50 ng/μL, RIN value > 7.0, and total RNA amount > 1 μg. Two rounds of purification were performed using oligo(dT) magnetic beads (Dynabeads Oligo (dT), #25‐61005, Thermo Fisher, USA) to specifically capture PolyA‐tailed mRNA. The mRNA was fragmented at 94°C for 5–7 min using a magnesium ion‐based fragmentation kit (NEBNext Magnesium RNA Fragmentation Module, cat.E6150S, USA). cDNA was synthesized using reverse transcriptase (Invitrogen SuperScript II Reverse Transcriptase, #1896649, CA, USA), followed by second‐strand synthesis using DNA Polymerase I from *E. coli* (NEB, #m0209, USA) and RNase H (NEB, #m0297, USA) to convert the DNA–RNA hybrid into double‐stranded DNA, incorporating dUTP Solution (Thermo Fisher, #R0133, CA, USA) and filling in the ends. An A‐base was added to each end, and adapters with a T‐base were ligated. The fragments were then purified using magnetic beads. The second strand was digested using UDG enzyme (NEB, cat.m0280, MA, USA), followed by PCR amplification with the following conditions: initial denaturation at 95°C for 3 min, 8 cycles of denaturation at 98°C for 15 s, annealing at 60°C for 15 s, and extension at 72°C for 30 s, with a final extension at 72°C for 5 min. This resulted in a library of 300 ± 50 bp fragments. Finally, paired‐end sequencing was performed using the illumina Novaseq 6000 (LC Bio Technology CO. Ltd. Hangzhou, China) in PE150 mode. All volcano plots and heatmaps were generated online using the OmicStudio platform (https://www.omicstudio.cn/). Raw sequencing data can be found in Table S1.

### 2.7 Immunohistochemistry

Tumor tissue from mice was prepared as 5 μm paraffin sections. After drying in a 60°C oven for 30 min, the sections were dewaxed by sequential washes in xylene I, II, and III for 10 min each. They were then hydrated through graded ethanol solutions (anhydrous ethanol I and II, followed by 95%, 80%, and 70% ethanol) for 2 min each, rinsed with distilled water, and subjected to heat‐induced antigen retrieval in pH 6.0 citrate buffer (Thermo Fisher, #005000) using a pressure cooker. Tissue areas were circled with an immunohistochemistry‐compatible waterproof pen to prevent reagent diffusion. Blocking was performed with 5% normal goat serum (homologous to the secondary antibody) at room temperature for 30 min. Primary antibodies against GPX4 (ABclonal, #A11243, rabbit source, 1:1000 dilution) and ACSL4 (ABclonal, #A20414, rabbit source, 1:1000 dilution) were applied, and the sections were incubated at 37°C for 2 h in a humidified chamber. After rinsing three times with PBS buffer (0.01 M, pH 7.4) for 5 min each, an HRP‐labeled goat anti‐rabbit secondary antibody (1:2000 dilution) was applied, and the sections were incubated at 37°C for 30 min. Following thorough washing with PBS, DAB chromogen (microscopically controlled development time) was used for color development. The sections were then counterstained with Mayer's hematoxylin for 30 s, differentiated with 1% hydrochloric acid in ethanol, and treated with PBS for bluing. Finally, the sections were dehydrated through graded ethanol, cleared with xylene, mounted with neutral balsam, and observed under an optical microscope for image acquisition.

### 2.8 Immunofluorescence

Mouse tumor tissue was prepared as 5 μm paraffin sections and processed through dewaxing and graded ethanol treatments (xylene I, II, III for 10 min each, followed by anhydrous ethanol I and II, 95%, 85%, and 75% ethanol for 2 min each). Antigen retrieval was performed by boiling the sections in pH 8.0 EDTA antigen retrieval solution (Servicebio, #G1207) for 8 min. After natural cooling, the sections were rinsed three times with phosphate‐buffered saline (PBS, 0.01 M, pH 7.4) for 5 min each and dried at 37°C. Blocking was done with 5% bovine serum albumin (BSA, Servicebio, #GC305010) at room temperature for 30 min. Primary antibodies against GPX4 (ABclonal, #A11243, 1:500 dilution), ACSL4 (ABclonal, #A20414, 1:1000 dilution), and CYP1B1 (ABclonal, #A1377, 1:200 dilution) were applied, and the sections were incubated overnight at 4°C in a humidified chamber. The next day, after thorough washing with PBS (three times, 5 min each), a Cy3‐labeled goat anti‐rabbit secondary antibody (1:1000 dilution, Servicebio) was applied, and the sections were incubated in the dark at 37°C for 1 h. Following PBS rinses, the sections were stained with DAPI nuclear stain (Servicebio, #G1012) in the dark at room temperature for 10 min. They were then treated with Autofluorescence quencher (Servicebio, #G1221) in the dark at room temperature for 5 min, rinsed with running water for 10 min to remove residual reagents, and finally mounted with an anti‐fade mounting medium (Servicebio). Images were acquired under a fluorescence microscope (Leica DMI3000 B), and fluorescence intensity was quantitatively analyzed using ImageJ software (Version 1.8.0).

### 2.9 Western Blotting (WB)

Total protein was extracted using RIPA lysis buffer (Beyotime, #P0013C). Equal amounts of total cell protein were separated by SDS‐polyacrylamide gel electrophoresis (SDS‐PAGE) and transferred to a PVDF membrane (Millipore, #IPVH00010). After blocking with 5% non‐fat milk (Sangon Biotech, #A600669) at room temperature for 1 h, the membranes were incubated with primary antibodies against CYP1B1 (ABclonal, #A1377), E‐Cadherin (CST, #4065), N‐Cadherin (CST, #4061), Vimentin (Abclonal, #A19607), FTH1 (CST, #3998), GPX4 (ABclonal, #A11243), ACSL4 (ABclonal, #A20414), NOX1 (ABclonal, #A8527), Phospho‐SP1‐T739 (Abclonal, #AP0231), Phospho‐NF‐kB p65/RelA‐S536 (Abclonal, #AP1294), Phospho‐c‐Jun‐S63 (Abclonal, #AP0105), TFAP2A (Abclonal, #A2294), P‐ERK1/2 (Abclonal, #AP0234), β‐actin (ABclonal, #AC026), and Histone (ABclonal, #A2348). The primary antibodies were incubated at 4°C overnight. The next day, after washing the membranes, they were incubated with horseradish peroxidase‐conjugated goat anti‐rabbit (Invitrogen, #31466) or goat anti‐mouse (PTG, #SA00001‐1) secondary antibodies at room temperature for 1 h. The membranes were then developed using ECL luminescent liquid (Millipore, #WBKLS0100), and images were captured using an imaging system. Image analysis was performed using ImageJ software.

### 2.10 Construction of Human Colorectal Cancer Cell Lines With CYP1B1 Knockdown and Overexpression

The overexpression and interference lentiviruses were purchased from Hanheng Biology with a titer of 1 × 10^9^ TU/mL. The overexpression plasmid was pHBLV‐CMV‐MCS‐EF1‐ZsGreen, and the interference plasmid was pHBLV‐U6‐MCS‐CMV‐ZsGreen. Based on the NCBI search for CYP1B1 (NM_000104.4), the OE_CYP1B1 sequence was as follows: F: 5′‐ACCGGTATGGGCACCAGCCTCAGCCC‐3′, R: 5′‐CCCGGGTTATTGGCAAGTTTCCTTGG‐3′. The sh_CYP1B1 sequence was: 5′‐CAGCATGATGCGCAACTTCTTCA‐3′, and the sh_NC sequence was: 5′‐TACATACGCTGCACTGACTTGC‐3′. The virus solution was added to the culture medium to infect cells in good growth condition for 8–12 h, followed by replacement with fresh complete medium containing puromycin (Biomiky). The selection process continued until approximately 90% of the cells expressed green fluorescence under microscopic observation. These cells were then used for subsequent experiments.

### 2.11 Prediction of Potential Drug Binding Targets

Download the two‐dimensional and three‐dimensional structures of the compound Calycosin (Compound CID: 5280448) from the PubChem database (https://pubchem.ncbi.nlm.nih.gov/), and import them into the SwissTarget Prediction database (https://swisstargetprediction.ch/). Set the search parameters to select the species “ *Homo sapiens* ” and predict potential binding targets of the compound. SwissTarget Prediction utilizes the 2D and 3D structures of known compounds to predict potential protein targets. The database includes interactions between 280,381 small molecules and 2686 targets. For each predicted target, SwissTarget Prediction provides a Probability score (> 0.5) to evaluate the likelihood of accurate prediction, which should be used as the filtering criterion.

### 2.12 Cellular Thermal Shift Assay (CETSA)

In HT29 and SW620 cells, treatment with DMSO and Calycosin was administered for 24 h. For site identification, the constructed H_CYP1B1 plasmids (including wild‐type WT, mutant MT1, and MT2) were transfected into HEK‐293 cells, respectively. After 24 h, the cells were treated with DMSO and Calycosin for another 24 h. Subsequently, 80 μL of cell suspension from each group was aliquoted into 8 PCR tubes of 0.2 mL each. The PCR machine (Thermo Fisher Scientific, VeritiPro) was set to 8 temperature points (35°C, 40°C, 45°C, 50°C, 55°C, 60°C, 65°C, and 70°C). The PCR tubes were heated in the machine for 3 min, immediately removed, and left to stand at room temperature for 3 min. Following this, 3 cycles of liquid nitrogen freeze–thaw were performed, and then the tubes were centrifuged at 12,000 rpm for 20 min at 4°C to collect the supernatant. After adding 1× SDS Loading buffer, the samples were boiled for 10 min, and 20 μL was used for the WB experiment. The primary antibodies used included: CYP1B1 (ABclonal, #A1377) and Flag‐DDDDK‐Tag (ABclonal, #AE092, 1:2000). ImageJ software (V1.8.0) was utilized for grayscale value analysis under different conditions.

### 2.13 Molecular Docking and Design of Amino Acid Mutation Sites

The protein ID for CYP1B1 (HUMAN_Q16678) was retrieved from the UniProt database (https://www.uniprot.org/). The protein structure of the target CYP1B1 (PDB_6IQ5) was downloaded from the RCSB database (https://www.rcsb.org). AutoDock (1.5.7) software was used to perform molecular docking between the Calycosin compound and the CYP1B1 protein. The docking results were visualized using PYMOL (2.5) software, and the intermolecular interactions, binding energy, and binding sites between the target and the small molecule were analyzed. Based on the molecular docking results, the key potential binding sites between Calycosin and the CYP1B1 protein were identified as ASN(N)‐265 and Gly(G)‐329. According to the principles of amino acid site mutation, the optimal mutation for ASN(N)‐265 is Thr(T)‐265, and the optimal mutation for Gly(G)‐329 is Ala(A)‐329.

### 2.14 Construction of Wild‐Type and Mutant H_CYP1B1 Overexpression Plasmids

Based on the optimal mutation sites, wild‐type H_CYP1B1, H_CYP1B1 (N265T), and H_CYP1B1 (G329A) plasmids were constructed. The PCDNA3.1‐MCS‐EF1‐ZsGreen (Genomeditech, #GM‐8630P1) was used as the vector plasmid for the target gene, and the *Escherichia coli* strain stbl3 (ThermoFisher, #G1036) was prepared for transformation. CYP1B1 primers were designed and synthesized, with homologous recombination sequences added to the 5′ end. The designed primer sequences were sent to a primer synthesis company (Suzhou Jinweizhi Biotechnology Co. Ltd) for synthesis. Finally, the PCDNA3.1‐H_CYP1B1–3 × flag‐EF1‐ZsGreen1 WT plasmid, PCDNA3.1‐H_CYP1B1 (N265T)‐3 × flag‐EF1‐ZsGreen1 Mut1 plasmid, and PCDNA3.1‐H_CYP1B1 (G329A)‐3 × flag‐EF1‐ZsGreen1 Mut2 plasmid were constructed.

### 2.15 Promoter Transfection and Luciferase Assay

To investigate the binding relationship between SP1 and the GPX4 promoter, a dual‐luciferase reporter gene assay was conducted. Initially, three binding sites for SP1 within the −2000 to 200 bp fragment of the GPX4 promoter were predicted using the Human Transcription Factor Database (Human TFDB, http://bioinfo.life.hust.edu.cn/HumanTFDB/). Fragments of the GPX4 promoter region (Full, Mut1, Mut2, and Mut3) were cloned into the pGL3‐basic vector, while SP1 was cloned into the pcDNA3.1–3xflag vector (Hanbio, China). HEK‐293 T cells were co‐transfected with pGL3‐Basic‐h‐GPX4 (Full, Mut1, Mut2, and Mut3) or pGL3‐Basic (NC) along with pcDNA3.1–3xflag‐h‐SP1 or pcDNA3.1–3xflag and the pRL‐TK Renilla luciferase vector. After 48 h, cell lysates were extracted, and firefly and Renilla luciferase activities were measured using the Dual‐Luciferase Reporter Assay Kit (HB‐DLR‐100, Hanbio, China). Luminescence signals were detected using a multifunctional microplate reader (SuPerMax3100, Shanghai Shanpu Biotechnology Co. Ltd., China). The experiment was repeated three times.

### 2.16 Statistical Analysis

Statistical analysis was performed using GraphPad Prism v8.0 (GraphPad Software, La Jolla, CA, USA, www.graphpad.com). Data are presented as mean ± standard error of the mean (Mean ± SEM). *p* values and significance were determined using two‐tailed t‐tests, One‐Way ANOVA, and Two‐Way ANOVA.

## 3 Results

### 3.1 Calycosin Effectively Inhibits Subcutaneous Tumor Growth in Mice With Colorectal Cancer

**FIGURE 1:**
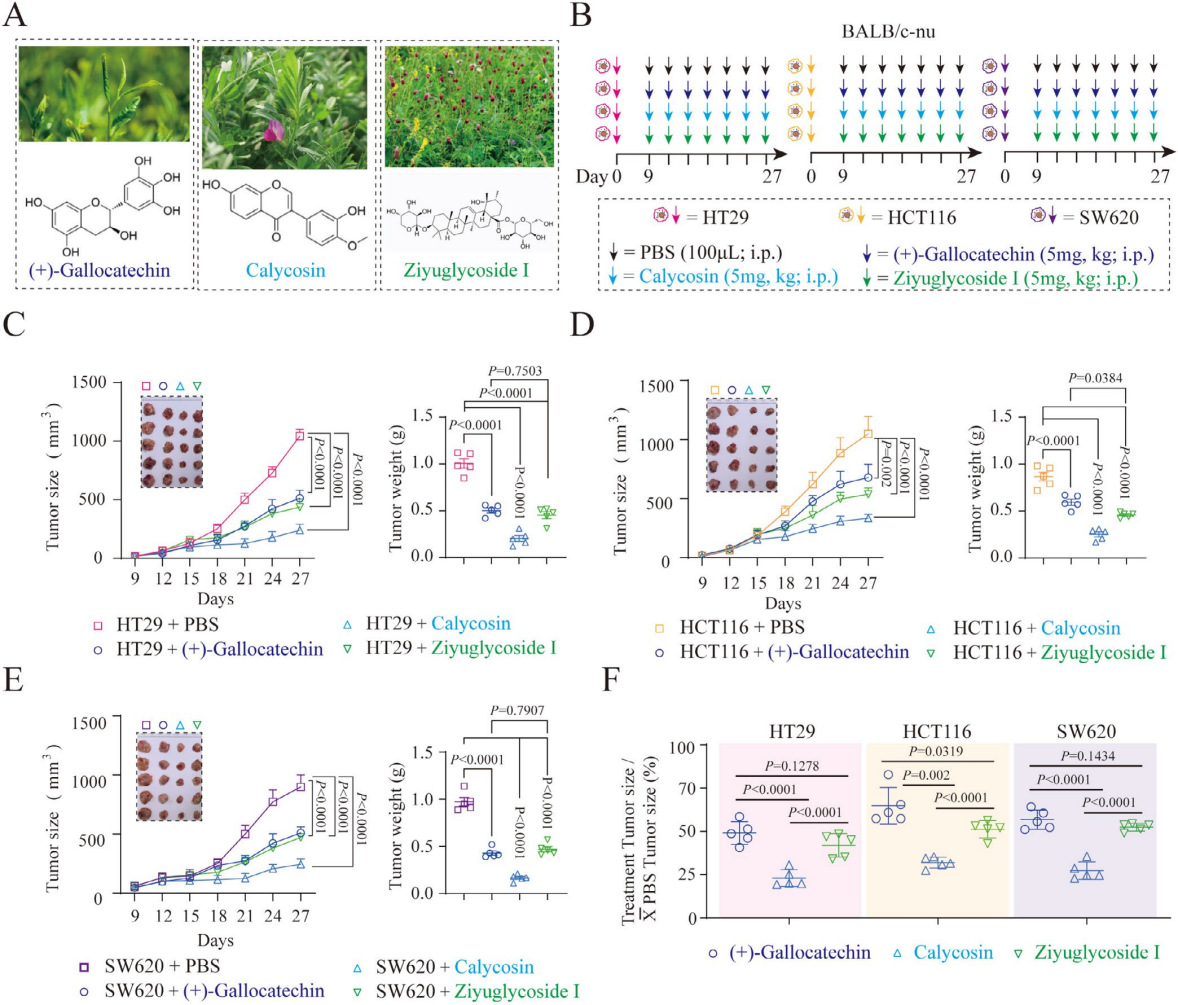
Therapeutic effects of three small‐molecule compounds from traditional Chinese medicine on subcutaneous colorectal cancer mouse models. (A) Schematic representation of the structures of the three small‐molecule compounds derived from traditional Chinese medicine. (B) Flowchart illustrating the construction and treatment process of the subcutaneous colorectal cancer (HT29, HCT116, SW620) mouse models. (C) Line graph depicting tumor volume and bar chart showing tumor weight in subcutaneous colorectal cancer (HT29) mouse models. (D) Line graph illustrating tumor volume and bar chart representing tumor weight in subcutaneous colorectal cancer (HCT116) mouse models. (E) Line graph showing tumor volume and bar chart displaying tumor weight in subcutaneous colorectal cancer (SW620) mouse models. (F) Bar chart demonstrating the relative changes in tumor volume area in response to treatment with the three small‐molecule compounds from traditional Chinese medicine across all colorectal cancer mouse models. Each data point signifies an individual mouse. Data are presented as mean ± standard error of the mean (SEM). *p* values and significance were determined using two‐tailed *t*‐tests (C–E) and One‐Way ANOVA (C–F).

To screen for small molecule drugs effective in the treatment of colorectal cancer, we constructed subcutaneous tumor mouse models of human colorectal cancer (HT29, HCT116, SW620) (Figure 1A,B) and examined the therapeutic effects of three small molecule compounds derived from traditional Chinese medicine: (+)‐Gallocatechin, Calycosin, and Ziyuglycoside I.

The results indicated that all three compounds, administered at the same dose (5 mg/kg) via intraperitoneal injection three times per day, exhibited therapeutic functionality in the subcutaneous mouse models of colorectal cancer, albeit with varying degrees of efficacy. Specifically, in the HT29 subcutaneous mouse model, Calycosin demonstrated the most pronounced inhibition of tumor growth, significantly reducing both tumor volume and mass (Figure 1C), while no significant difference was observed between the (+)‐Gallocatechin and (+)‐Gallocatechin treatment groups. In the HCT116 subcutaneous mouse model, Calycosin also exhibited the most significant inhibitory effect on tumor growth, but the efficacy of Ziyuglycoside I was superior to that of (+)‐Gallocatechin (Figure 1D). Similarly, in the SW620 subcutaneous mouse model, Calycosin exhibited the most prominent therapeutic effect, superior to both (+)‐Gallocatechin and Ziyuglycoside I, with no significant difference in efficacy between the latter two groups (Figure 1E,F).

After 18‐day administration of 5 mg/kg Calycosin, (+)‐Gallocatechin, and (+)‐Gallocatechin to BALB/c‐nu mice, compared to the PBS group, no significant change in body weight was seen (Figure S1A,B). Consistently, no obvious pathological changes occurred in the mice's organs (heart, liver, spleen, lung, kidney, and brain), and no significant difference in blood ALT and AST levels was observed (Figure S1C,D). Thus, 5 mg/kg Calycosin, (+)‐Gallocatechin, and (+)‐Gallocatechin are safe for BALB/c‐nu mice.

### 3.2 Calycosin Inhibits Migration, EMT, and Promotes Apoptosis of Colorectal Cancer Cells In Vitro

**FIGURE 2:**
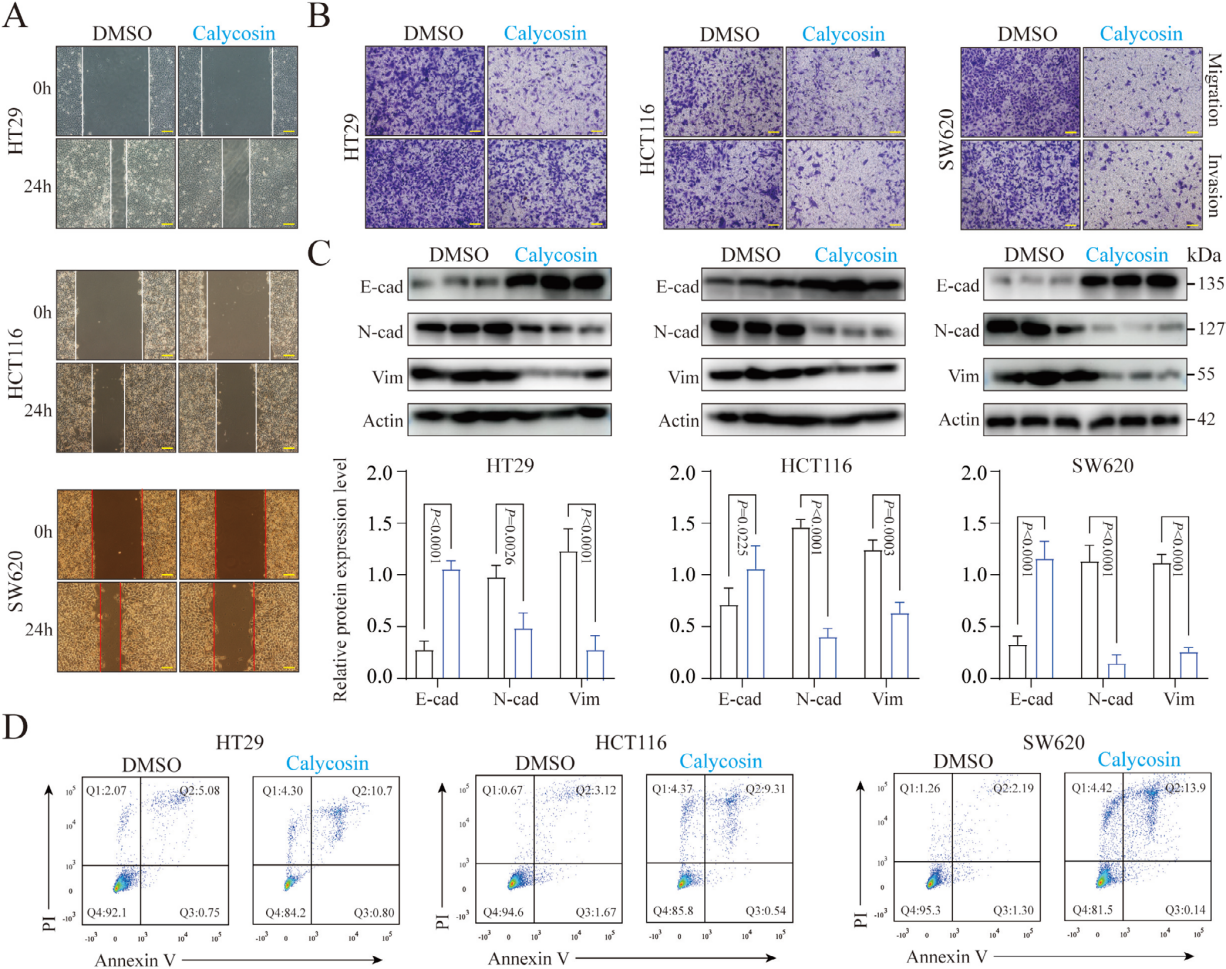
Effects of Calycosin on migration, invasion, EMT, and apoptosis of colorectal cancer cells in vitro. (A) Cell scratch assay to evaluate the impact of Calycosin on the migration of colorectal cancer cells. Scale bar = 100 μm. (B) Transwell assay assessing the influence of Calycosin on the migration and invasion of colorectal cancer cells. Scale bar = 50 μm. (C) Western blot (WB) analysis examining the effect of Calycosin on the expression of EMT‐related proteins in colorectal cancer cells. (D) Flow cytometry detecting the effect of Calycosin on the apoptosis of colorectal cancer cells. Data are presented as mean ± standard error of the mean (Mean ± SEM). *p* values and significance were determined by Two‐Way ANOVA (C).

To evaluate the effects of Calycosin on the function of colorectal cancer cells in vitro, our experimental results demonstrated that the half‐maximal inhibitory concentrations (IC50) of Calycosin against HT29, HCT116, and SW620 cell lines were 124.6, 132.7, and 113.8 μM, respectively (Figure S2A). Consequently, we treated human colorectal cancer HT29, HCT116, and SW620 cells with 100 μM Calycosin for 24 h. Scratch assay results indicated that Calycosin effectively inhibits the migration of human colorectal cancer cells (Figure 2A), particularly SW620 cells (Figure S2B). Consistent with this, Transwell assay results showed that Calycosin treatment for 24 h also suppressed the migration and invasion of human colorectal cancer cells (Figures 2B and S2C,D). Since EMT is a crucial factor driving tumor cell migration and invasion (Yang et al. 2024), we investigated the impact of Calycosin on the expression of EMT‐related proteins in tumor cells. The results demonstrated that Calycosin enhances the expression of E‐Cadherin while suppressing N‐Cadherin and Vimentin protein expression, indicating that Calycosin inhibits EMT in tumor cells (Figure 2C). Previous research has revealed that Calycosin promotes tumor cell apoptosis (Zhang et al. 2024), which directly affects tumor cell function (Lv et al. 2023). Therefore, we used flow cytometry to assess the effect of Calycosin on colorectal cancer cell apoptosis. The results showed that Calycosin induces apoptosis in human colorectal cancer cells (Figure 2D), with SW620 being the most sensitive (Figure S2E).

### 3.3 Calycosin Activates the Expression of Ferroptosis‐Related Proteins in Colorectal Cancer Cells

**FIGURE 3:**
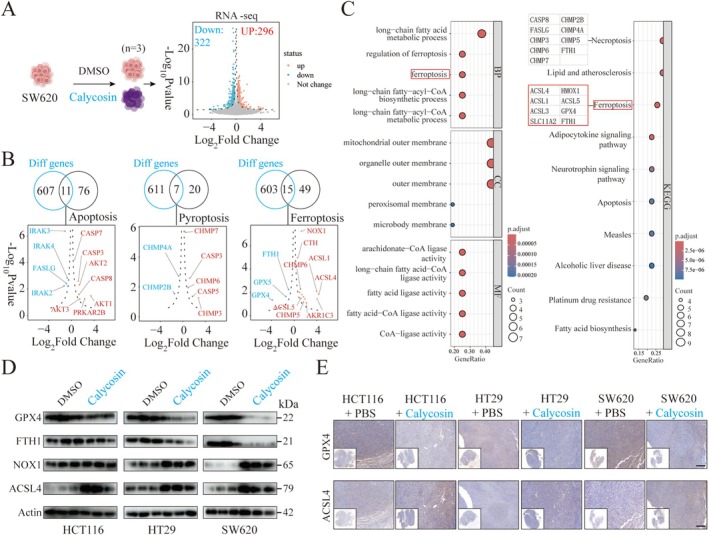
The influence of Calycosin on the expression of genes related to colorectal cancer cell death and its identification. (A) Results of whole‐genome transcriptome sequencing after treating SW620 cells (*n* = 3) with DMSO and Calycosin (100 μM) for 24 h. The volcano plot of differentially expressed genes is shown on the right, with upregulated genes in red and downregulated genes in blue. (B) Volcano plot depicting the expression of genes related to apoptosis, necrosis, and ferroptosis in SW620 cells. (C) Bubble chart showing GO and KEGG pathway enrichment analysis of genes related to apoptosis, necrosis, and ferroptosis. (D) Protein expression levels of GPX4, FTH1, NOX1, and ACSL4 after treating HCT116, HT29, and SW620 cells with DMSO and Calycosin (100 μM) for 24 h. (E) Immunohistochemical detection of GPX4 and ACSL4 expression in three types of subcutaneous colorectal cancer mouse tumor tissues. Scale bar = 100 μm.

To further analyze the impact of Calycosin on the relevant proteins and pathways involved in colorectal cancer cell death, we treated SW620 cells with DMSO and Calycosin (100 μM) for 24 h before conducting whole‐genome transcriptome sequencing (Table S1). The results indicated that, compared to DMSO, 296 genes were upregulated and 322 genes were downregulated in the Calycosin group (|Log2FC| > 0.5, *p* < 0.05) (Figure 3A and Table S2). Given the diverse types of cell death regulated by drugs, we searched the GSEA database using apoptosis, necrosis, and ferroptosis as key search terms. After screening, we obtained 87 apoptosis‐related genes, 27 necrosis‐related genes, and 64 ferroptosis‐related genes (Table S3). The results showed that after treating SW620 cells with Calycosin, the most significant change was observed in the number of ferroptosis‐related genes.

Specifically, pro‐ferroptosis proteins ACSL1, ACSL4, and NOX1 genes were upregulated, while anti‐ferroptosis proteins GPX4 and FTH1 genes were downregulated (Figure 3B). Similarly, GO and KEGG pathway enrichment analyses were performed on the changed apoptosis, necrosis, and ferroptosis‐related genes. The results revealed that these genes were mainly enriched in ferroptosis‐related signaling pathways (Figure 3C). Consistent with this, after treating HCT116, HT29, and SW620 cells with Calycosin (100 μM) for 24 h, we found that the ferroptosis proteins GPX4 and FTH1 were downregulated, while NOX1 and ACSL4 were upregulated in all three cell lines (Figure 3D). Among these, the most pronounced alterations in ferroptosis‐related proteins were observed in SW620 cells (Figure S3A). Similarly, after stimulating SW620 cells with Calycosin, treatment with the ferroptosis inhibitor Ferrostatin‐1 (10 μM, 2 h) was performed. The results demonstrated that Ferrostatin‐1 treatment significantly suppressed the expression of NOX1 and ACSL4 proteins while upregulating GPX4 and FTH1 expression (Figure S3B). Concurrently, flow cytometry analysis revealed that Ferrostatin‐1 treatment markedly inhibited Calycosin‐induced apoptosis (Figure S3C). These findings suggest that Calycosin may exert its effects on tumor cell function through the modulation of ferroptosis.

Additionally, in vivo detection of GPX4 and ACSL4 expression in subcutaneous mouse models of HT29, HCT116, and SW620 from PBS and Calycosin treatment groups showed that in the Calycosin treatment group, GPX4 was downregulated and ACSL4 was upregulated in all three mouse tumor tissues (Figure 3E).

### 3.4 Binding of Calycosin to the Gly‐329 Site of CYP1B1

**FIGURE 4:**
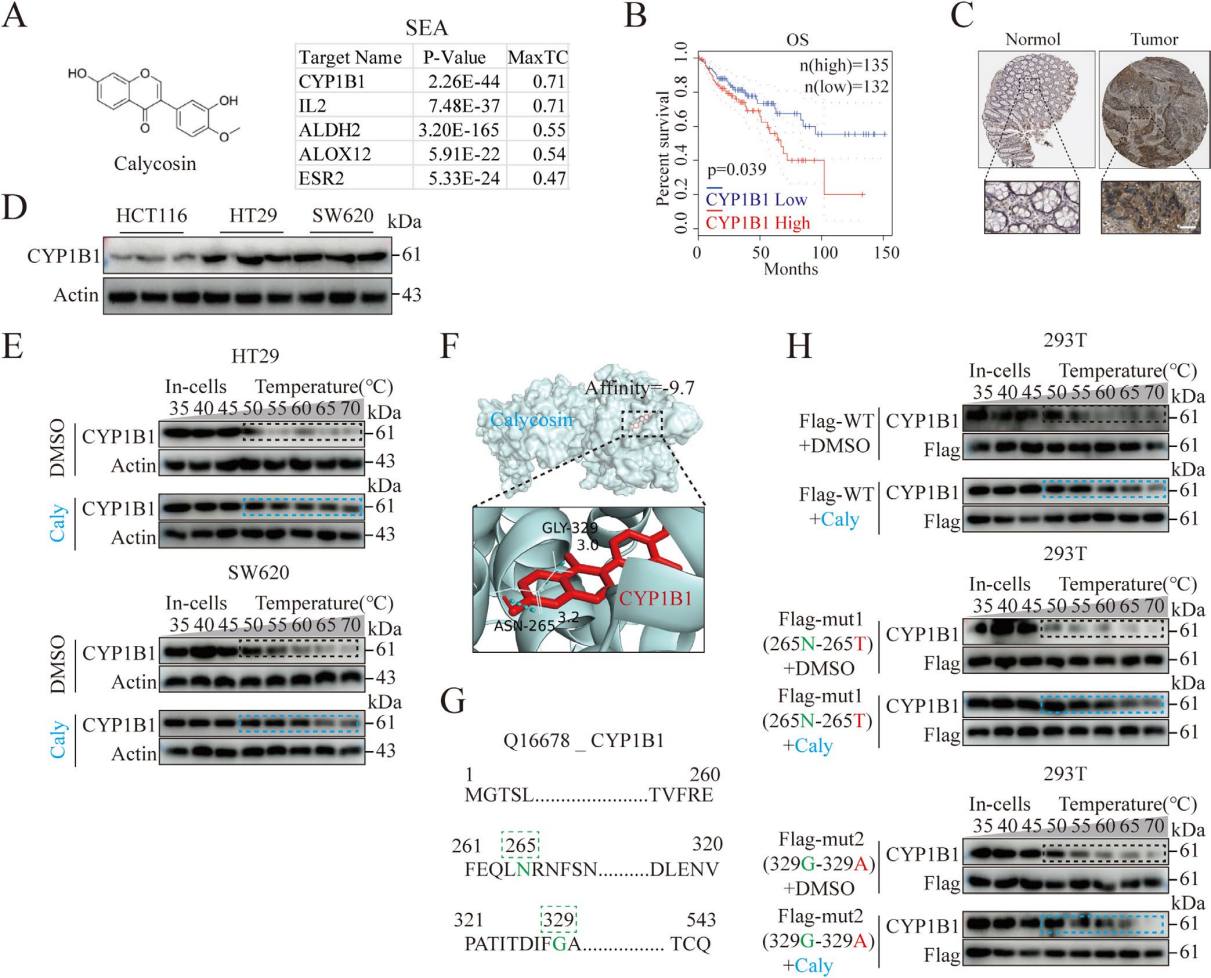
Identification of potential binding targets and sites of Calycosin. (A) Structural formula of Calycosin and its potential binding targets. (B) Prognostic curves for CYP1B1 gene in CRC from the GEPIA database. (C) Expression of CYP1B1 protein in normal and tumor tissues of the colon and rectum from the HPA database. Scale bar = 50 μm. (D) CYP1B1 protein expression levels in HCT116, HT29, and SW620 cells. (E) CETSA assessment of the thermal stability of CYP1B1 after treatment with Calycosin (100 μM) for 24 h in HT29 and SW620 cells. (F) Molecular docking calculation of the binding energy between Calycosin and CYP1B1. (G) Amino acid sequence of CYP1B1 protein from the UniProt database. (H) Effect of Calycosin on the thermal stability of wild‐type (WT) H_CYP1B1 and overexpressed mutant H_CYP1B1 (N265T) Mut1, H_CYP1B1 (G329A) Mut2 in HEK‐293 cells.

To explore the molecular mechanism by which Calycosin regulates colorectal cancer cell death, it is necessary to identify the target of Calycosin's action. Since no existing literature has explicitly reported the direct molecular targets of calycosin in ferroptosis regulation, this study systematically screened potential binding targets based on the three‐dimensional protein structures of calycosin retrieved from the Similarity Ensemble Approach (SEA) database. Among the potential targets identified, CYP1B1, IL2, ALDH2, ALOX12, and ESR2 were found, with CYP1B1 and IL2 having the highest binding probability (MaxTC, where MaxTC = 1 represents 100% binding) (Figure 4A). Further screening using the GEPIA database revealed that only CYP1B1 had a significant negative correlation with the overall survival (OS) prognosis of CRC (Figure 4B). Additionally, the HPA database indicated high expression of CYP1B1 in colorectal cancer patients (Figure 4C), suggesting that highly expressed CYP1B1 may be a key gene affecting the prognosis of colorectal cancer.

To confirm CYP1B1 as the binding target of Calycosin, we selected HT29 and SW620 cells, which express the highest levels of CYP1B1 (Figure 4D). The Cellular Thermal Shift Assay (CETSA) was used to detect the binding of Calycosin (100 μM) to CYP1B1 protein in live cells. The results showed that in HT29 and SW620 cells treated with Calycosin for 24 h, CYP1B1 protein could resist temperature‐induced protein degradation (Figure 4E), indicating that Calycosin can bind to CYP1B1 and maintain its thermal stability.

Consistent with this, molecular docking experiments revealed a high binding affinity between Calycosin and CYP1B1 (Affinity = −9.7) (Figure 4F). ASN(N)‐265 and Gly(G)‐329 were identified as potential binding sites for Calycosin and CYP1B1. Based on the principle of amino acid site mutation, which states that mutations should not affect protein structure and function (Rodrigue et al. 2010), we mutated ASN(N)‐265 and Gly(G)‐329 to Thr(T)‐265 and Ala(A)‐329, respectively (Figure 4G). We constructed wild‐type (WT) H_CYP1B1 and mutant overexpression plasmids H_CYP1B1 (N265T) Mut1 and H_CYP1B1 (G329A) Mut2. These plasmids were transfected into HEK‐293 cells to test the thermal stability of CYP1B1 protein after Calycosin treatment.

The results showed that both wild‐type (WT) CYP1B1 and CYP1B1(N265T) proteins maintained their thermal stability after treatment with Calycosin (100 μM), while CYP1B1(G329A) did not. This suggests that Gly‐329 may be a critical binding site for Calycosin and CYP1B1 (Figure 4H).

### 3.5 Knockdown and Overexpression of CYP1B1 Can Respectively Inhibit and Promote Colorectal Cancer Cell Functions

**FIGURE 5:**
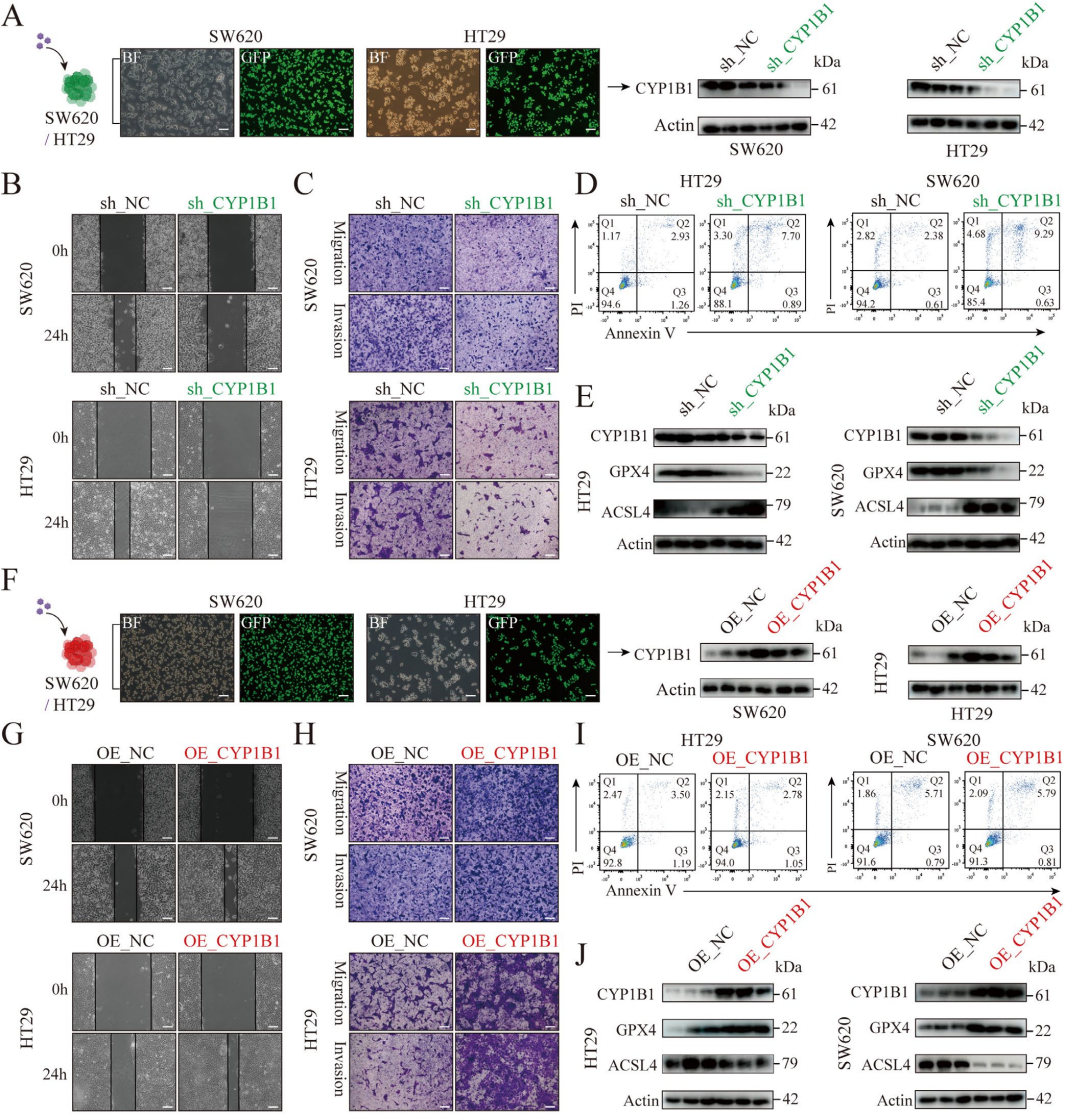
The impact of CYP1B1 on the in vitro functions of colorectal cancer cells. (A) Process and identification of stably knocking down CYP1B1 in SW620 and HT29 cell lines. The left panel shows the efficiency of lentiviral infection in SW620 and HT29, while the right panel displays the protein expression of CYP1B1 in the control (NC) and CYP1B1 knockdown (sh_CYP1B1) groups. (B) Cell scratch assay to assess the effect of CYP1B1 knockdown on the migration ability of SW620 and HT29 cells. Scale bar = 100 μm. (C) Transwell assay to evaluate the impact of CYP1B1 knockdown on the migration and invasion of SW620 and HT29 cells. Scale bar = 50 μm. (D) Flow cytometry analysis to determine the effect of CYP1B1 knockdown on the apoptosis of SW620 and HT29 cells. (E) WB analysis to examine the protein expression of CYP1B1, GPX4, and ACSL4 in SW620 and HT29 cells after CYP1B1 knockdown. (F) Process and identification of stably overexpressing CYP1B1 in SW620 and HT29 cell lines. The left panel illustrates the efficiency of lentiviral infection in SW620 and HT29, while the right panel shows the protein expression of CYP1B1 in the control (NC) and CYP1B1 overexpression (OE_CYP1B1) groups. (G) Cell scratch assay to investigate the effect of CYP1B1 overexpression on the migration ability of SW620 and HT29 cells. Scale bar = 100 μm. (H) Transwell assay to assess the impact of CYP1B1 overexpression on the migration and invasion of SW620 and HT29 cells. Scale bar = 50 μm. (I) Flow cytometry analysis to determine the effect of CYP1B1 overexpression on the apoptosis of SW620 and HT29 cells. (J) WB analysis to examine the protein expression of CYP1B1, GPX4, and ACSL4 in SW620 and HT29 cells after CYP1B1 overexpression.

To analyze the impact of CYP1B1 on colorectal cancer cell functions, we constructed SW620 and HT29 cell lines with stable CYP1B1 knockdown (sh_CYP1B1) (Figures 5A and S4A). The results showed that compared to the NC group, the migration and invasion abilities of cells in the sh_CYP1B1 group were inhibited (Figure 5B,C). Additionally, flow cytometry analysis revealed that knockdown of CYP1B1 increased the number of dead SW620 and HT29 cells (Figures 5D and S4B). Consistent with this, knockdown of CYP1B1 downregulated GPX4 protein and upregulated ACSL4 protein in SW620 and HT29 cells (Figures 5E and S4C).

Furthermore, we also constructed SW620 and HT29 cell lines with CYP1B1 overexpression (OE_CYP1B1) (Figures 5F and S4D). The results indicated that compared to the NC group, the migration and invasion abilities of cells in the OE_CYP1B1 group were enhanced (Figure 5G,H). However, flow cytometry analysis found that overexpression of CYP1B1 had no significant effect on the death of SW620 and HT29 cells (Figures 5I and S4E). Meanwhile, overexpression of CYP1B1 promoted GPX4 and inhibited ACSL4 protein expression in SW620 and HT29 cells (Figures 5J and S4F). To investigate the effects of calycosin on SW620 and HT29 cells overexpressing CYP1B1, we treated these cells with calycosin and observed that calycosin similarly inhibited the migration and invasion of both SW620 and HT29 cells (Figure S4G,H).

In summary, CYP1B1 may be a key target affecting the migration, invasion, and ferroptosis of colorectal cancer cells.

### 3.6 CYP1B1 and Calycosin Effects on Colorectal Cancer Cell Function in a Mouse Model and Their Interaction Analysis

**FIGURE 6:**
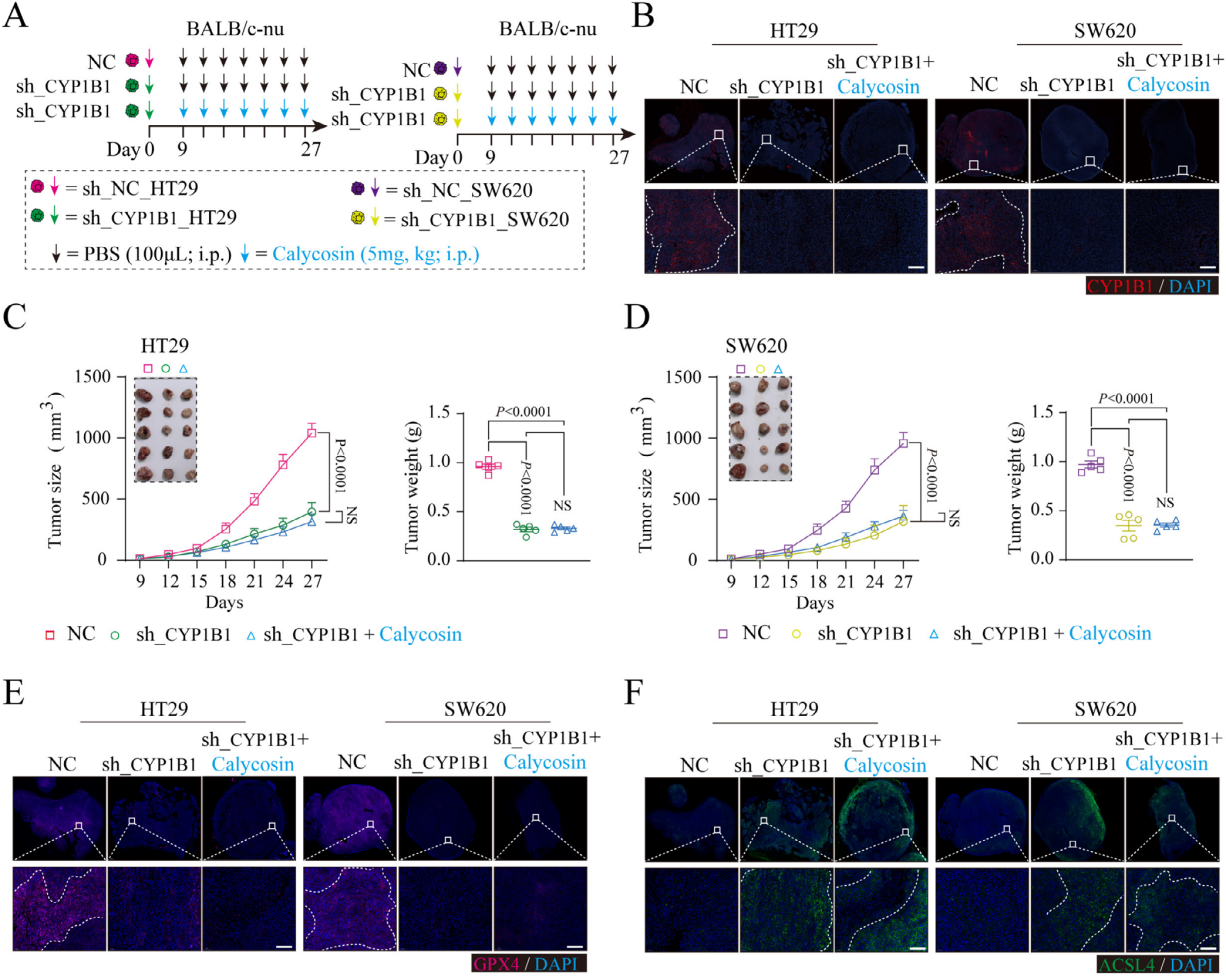
The impact of CYP1B1 on subcutaneous tumor growth in colorectal cancer mice. (A) Establishment of subcutaneous mouse models of human colorectal cancer (HT29, SW620) with stable CYP1B1 knockdown (sh_CYP1B1) and subsequent treatment with Calycosin. (B) Immunofluorescence detection of CYP1B1 expression in subcutaneous tumor tissues of human colorectal cancer mice in three groups (NC, sh_CYP1B1, sh_CYP1B1 + Calycosin), Scale bar = 100 μm. (C, D) Line charts of tumor volume and bar charts of tumor weight in subcutaneous tumor mice with stable CYP1B1 knockdown in HT29 (C) and SW620 (D), with each point representing an individual mouse. (E, F) Immunofluorescence detection of GPX4 (E) and ACSL4 (F) expression levels in tumor tissues of subcutaneous tumor mice with stable CYP1B1 knockdown in HT29 and SW620, Scale bar = 50 μm. Data are presented as Mean ± SEM, and *p* values and significance were determined by two‐tailed Student's *t*‐test (C, D) and One‐Way ANOVA (C, D).

To further investigate the effects of CYP1B1 on colorectal cancer cell function in vivo and to determine whether CYP1B1 is a key regulatory target of Calycosin, we used stably CYP1B1‐knockdown (sh_CYP1B1) HT29 and SW620 cell lines to establish subcutaneous mouse models, respectively. Simultaneously, Calycosin treatment was administered in the sh_CYP1B1 group of mice to evaluate whether CYP1B1 is a crucial target for Calycosin's action (Figure 6A). The results showed that compared to the NC group, CYP1B1 protein was effectively knocked down in the sh_CYP1B1 group (Figure 6B). Tumor size and weight data indicated that knocking down CYP1B1 could effectively inhibit the growth of SW620 and HT29 cells in vivo compared to the NC group (Figure 6C,D). However, there was no significant difference in tumor size and weight between the sh_CYP1B1 and sh_CYP1B1 + Calycosin groups (Figure 6C,D). This suggests that after knocking down CYP1B1, Calycosin may have lost its regulatory target, thus unable to modulate tumor growth. Additionally, the expression of GPX4 and ACSL4 was examined in tumor tissues. The results demonstrated that knocking down CYP1B1 inhibited GPX4 expression and promoted ACSL4 expression in SW620 and HT29 subcutaneous mouse tumor tissues (Figure 6E,F). No significant difference was observed in GPX4 and ACSL4 expression between the sh_CYP1B1 and sh_CYP1B1 + Calycosin groups (Figure 6E,F).

### 3.7 CYP1B1 Promotes GPX4 Expression via Activation of the AKT/SP‐1 Signaling Pathway

**FIGURE 7:**
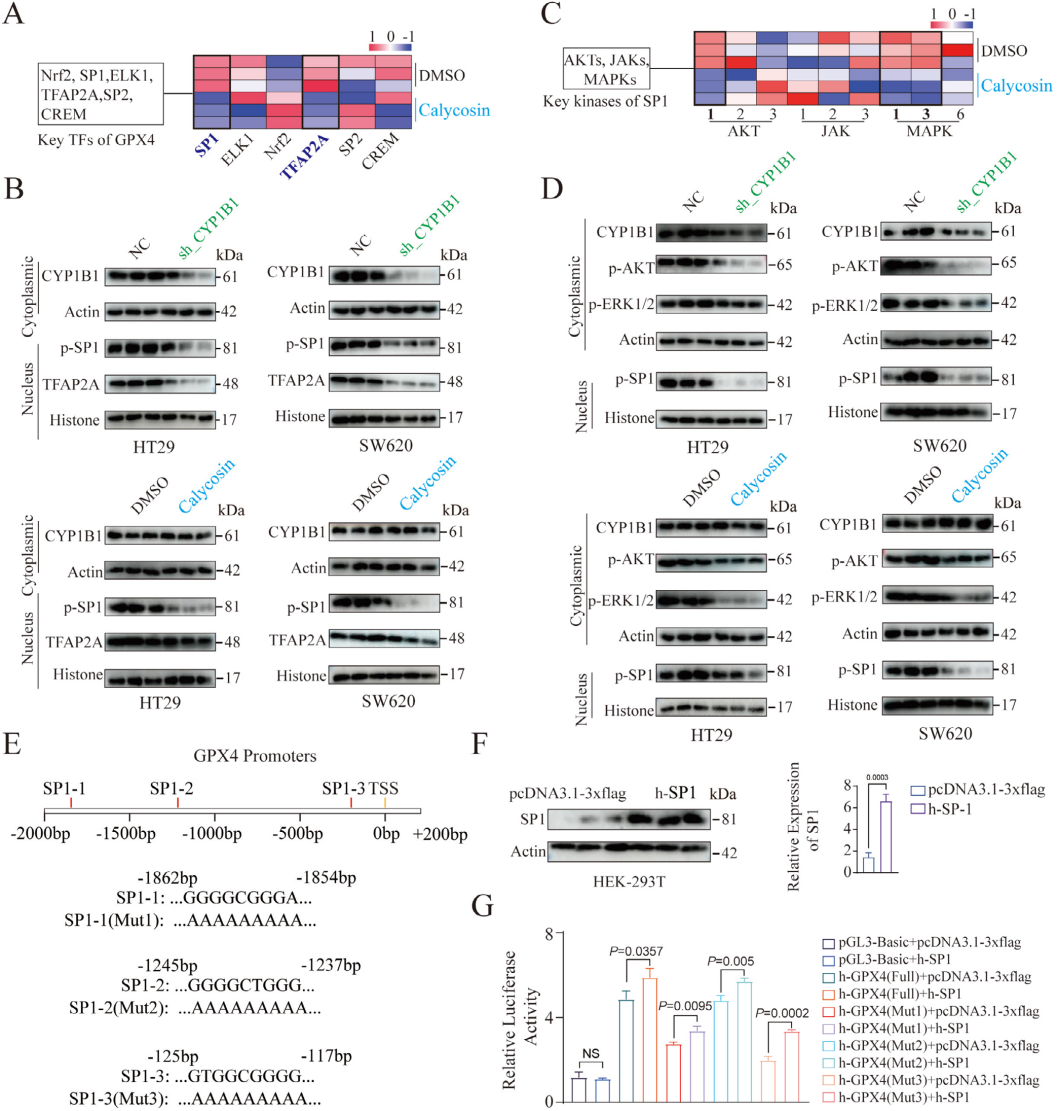
Molecular mechanism of CYP1B1 regulating GPX4 expression. (A) Expression of key transcription factors regulating GPX4 in transcriptome sequencing data from SW620 cells treated with DMSO and Calycosin. (B) Protein expression of CYP1B1 in the cytoplasm and p‐SP1 and TFAP2A in the nucleus of HT29 and SW620 cells after CYP1B1 knockdown and Calycosin treatment. (C) Expression of key kinases AKTs, JAKs, and MAPKs regulating GPX4 in transcriptome sequencing data from SW620 cells treated with DMSO and Calycosin. (D) Protein expression of CYP1B1, p‐AKT1, p‐ERK1/2 in the cytoplasm, and p‐SP1 in the nucleus of HT29 and SW620 cells after CYP1B1 knockdown and Calycosin treatment. (E) Three potential binding sites of SP1 on the GPX4 promoter. (F) Protein expression levels of SP‐1 after transfection of wild‐type and h‐SP1 overexpressing plasmids into HEK‐293 T cells. (G) Luciferase activity detected after transfection of wild‐type/h‐SP1 overexpressing plasmids into HEK‐293 T cells expressing the full‐length/mutant GPX4 promoter. Data are presented as mean ± standard error of the mean (Mean ± SEM). *p* values and significance were determined by two‐tailed *t*‐test (F, G).

Previous studies have reported that CYP1B1, which is highly expressed in colorectal cancer, can induce tumor cell resistance to ferroptosis by promoting the ubiquitination and degradation of ACSL4 (Chen et al. 2023). This is consistent with the results obtained in this study, both in vitro and in vivo (Figures 5 and 6). However, the molecular mechanisms by which CYP1B1 regulates GPX4 protein expression and how Calycosin modulates ferroptosis in tumor cells through CYP1B1 remain elusive and require further investigation. CYP1B1 is a metabolic enzyme that has been reported to affect the expression of kinases such as AKT and MAPK, as well as their downstream transcription factors, thereby regulating tumor cell activity (Kwon et al. 2016). Utilizing transcriptome sequencing data from SW620 cells treated with DMSO and Calycosin, we analyzed the expression of key transcription factors Nrf2, SP1, ELK1, TFAP2A, SP2, and CREM, which regulate GPX4. We found that Calycosin can inhibit the expression of SP1 and TFAP2A (Figure 7A). Consistent with this, knockdown of CYP1B1 in HT29 and SW620 cells also inhibited the expression of p‐SP1 and TFAP2A in the nucleus (Figures 7B and S5A). Treatment with Calycosin did not significantly alter CYP1B1 expression in HT29 and SW620 cells but inhibited the expression of p‐SP1 and TFAP2A in the nucleus, with a stronger inhibitory effect on p‐SP1 (Figures 7B and S5B).

Further analysis of key kinases regulating SP1 revealed that Calycosin can inhibit the expression of AKT1, MAPK1 (ERK2), and MAPK3 (ERK1) (Figure 7C). Similarly, both CYP1B1 knockdown and Calycosin treatment inhibited the phosphorylation levels of AKT1 and ERK1/2, suppressing p‐SP1 expression in the nucleus (Figure 7D). Here, phosphorylated AKT exhibited higher inhibitory efficiency (Figure S5C,D). To analyze the regulatory role of AKT on SP1 and GPX4, HT29 and SW620 cells were treated with the AKT inhibitor MK‐2206. The results demonstrated that inhibition of AKT phosphorylation significantly reduced the expression levels of p‐SP1 and GPX4 (Figure S5E).

Additionally, based on the identification of three potential binding sites for SP1 on the GPX4 promoter, we constructed corresponding mutants and an h‐SP‐1 overexpression plasmid (Figure 7E,F). The results indicated that the promoter regions Mut1: −1862 ~ −1854 and Mut3: −125 ~ −117 are critical for SP1 binding and activation of GPX4 (Figure 7G).

## 4 Discussion

Colorectal cancer, as the third most common malignancy in the world, has been a focal point of research in optimizing its treatment strategies (Dekker et al. 2019). Traditional chemotherapy drugs often accompany significant toxic side effects, severely affecting the quality of life of patients, while immune checkpoint inhibitors demonstrate primary drug resistance in some patients (Fan et al. 2021). Therefore, exploring new therapeutic strategies and medications has become an urgent research need.

This study systematically elucidates the molecular mechanism of Calycosin in the treatment of colorectal cancer through integrated in vitro and in vivo experiments, providing a new theoretical basis for targeted therapy of colorectal cancer. As a natural small‐molecule compound of flavonoids from traditional Chinese medicine, Calycosin exhibits significant pharmacological activity in the field of anti‐tumor therapy (Zhao et al. 2016). Previous studies have indicated that Calycosin, by targeting ERβ, up‐regulating PTEN, and inhibiting the PI3K/Akt signaling pathway, promotes apoptosis and suppresses the progression of colorectal cancer (Zhu et al. 2022). Nevertheless, the exact mechanism underlying Calycosin‐induced colorectal cancer cell death remains to be fully elucidated. By constructing a subcutaneous mouse model of human colorectal cancer, this study found that Calycosin can effectively inhibit tumor growth and suppress the migration, invasion, and epithelial‐mesenchymal transition (EMT) of human colorectal cancer cells in vitro, promoting ferroptosis. These results suggest that Calycosin may inhibit tumor progression and metastasis through multiple mechanisms. Transcriptome sequencing analysis further revealed the impact of Calycosin on genes related to colorectal cancer cell death. Calycosin affects the ferroptosis process of tumor cells by inhibiting GPX4 and promoting ACSL4 protein expression. Ferroptosis is a novel form of cell death that primarily affects tumor activity by regulating iron metabolism and lipid peroxidation‐related pathways (Jiang et al. 2021). We discovered that Calycosin promotes this process by regulating the expression of related proteins. Additionally, target prediction and molecular docking techniques identified CYP1B1 as a potential binding target of Calycosin. Cellular thermal shift assay (CETSA) further confirmed that Calycosin enhances the thermal stability of the CYP1B1 protein, while site‐directed mutagenesis experiments identified 329Gly as a critical binding site for Calycosin and CYP1B1. These findings provide molecular‐level evidence for understanding the mechanism of Calycosin.

Mechanistically, Calycosin promotes ferroptosis by binding to CYP1B1, inhibiting AKT activation, and reducing the binding of SP‐1 to the GPX4 gene promoter. The revelation of this mechanism not only explains the anti‐tumor activity of Calycosin but also provides a new molecular target for the treatment of colorectal cancer. Consistent with previous studies, it has been reported that CYP1B1‐derived 20‐HETE in colorectal cancer (CRC) upregulates FBXO10 expression by activating the protein kinase C (PKC) pathway, thereby promoting the ubiquitination and degradation of ACSL4 and ultimately inducing resistance to ferroptosis in tumor cells (C. Chen et al. 2023). Furthermore, in extrahepatic cholangiocarcinoma (ECC), aldo‐keto reductase family 1 member C1 (AKR1C1) reduces the protein stability of cytochrome P450 family member CYP1B1 through ubiquitin‐proteasome degradation, consequently triggering ferroptosis (Liu et al. 2025). These findings indicate that CYP1B1 is a critical protein influencing ferroptosis in tumors. Additionally, as a metabolic enzyme, CYP1B1 can modulate the expression of kinases such as AKT and MAPK, as well as their downstream transcription factors, thereby regulating tumor cell activity (Cicek et al. 2005).

In summary, the findings of this study not only offer new hope for the treatment of colorectal cancer patients but also open up a new path for the application of small‐molecule compounds from traditional Chinese medicine in the anti‐tumor field. With the further elucidation of Calycosin's mechanism of action, it is expected to become a new effective drug for the treatment of colorectal cancer, bringing better treatment effects and quality of life to patients. Furthermore, considering that CYP1B1 inhibitors can be clinically combined with PARP inhibitors to treat ovarian cancer (Xue et al. 2024), and given that CYP1B1 is the target of Calycosin, future studies should evaluate the therapeutic efficacy of Calycosin in combination with relevant chemotherapeutic or targeted agents for CRC. Such investigations would assess the synergistic potential of Calycosin, thereby providing a robust foundation for its clinical application as an antitumor agent.

## 5 Conclusion

This study elucidates that calycosin, a natural flavonoid, inhibits colorectal cancer (CRC) by targeting CYP1B1 to induce ferroptosis. It binds CYP1B1, suppresses AKT activation, and reduces SP‐1 binding to the GPX4 promoter, downregulating GPX4 while upregulating ACSL4. This promotes ferroptotic death, concurrently suppressing CRC migration, invasion, and EMT in vitro and tumor growth in vivo. Given CYP1B1's role in ferroptosis regulation and prior clinical applications, calycosin‐based combination therapies with conventional agents warrant exploration for CRC treatment. These findings provide a novel molecular foundation for CRC targeted therapy.

## Author Contributions

**Lihan Bie:** experimental design, data curation, formal analysis, methodology, writing original draft. **Xin Lei** and **Di Wu:** data curation, formal analysis, methodology, writing original draft. **Yang Zhang**, **Chengshan He**, **Luyao Liu**, and **Jiawei Zhou:** methodology, collected the information and revised and finalized the paper. **Xin Zhou**, **Yingying Lu**, and **Zheng Xu:** investigation, supervision, writing – review and editing. All authors read and approved the final manuscript.

## Funding

This work was supported by The Scientific Research Program of Shanghai Pudong New Area Health Commission (PW2022A‐22).

## Ethics Statement

The animal experiments were approved by the Medical Ethics Committee of the Seventh People's Hospital of Shanghai (Ethics Approval Number: 2025‐AR‐002).

## Conflicts of Interest

The authors declare no conflicts of interest.

## Supporting information


**Figure S1:** Effects of three small‐molecule compounds derived from traditional Chinese medicine on body weight, organ toxicity, and metabolic function in BALB/c‐nu mice. (A) Schematic diagram of the administration of Calycosin, (+)‐Gallocatechin and Ziyuglycoside I in BALB/c‐nu mice. (B) Line graph depicting changes in mouse body weight. (C) Gross photographs and pathological results of the heart, liver, spleen, lungs, kidneys, and brain. Histopathological examination of the heart, spleen, lungs, and kidneys was performed using hematoxylin and eosin (HE) staining, while liver tissue lesions were assessed via Oil Red O staining, and brain tissue lesions were evaluated using Nissl staining. Scale bar = 100 μm. (D) Expression levels of alanine transaminase (ALT) and aspartate aminotransferase (AST) in peripheral blood after model establishment. Data are presented as mean ± standard error of the mean (SEM). *p* values and significance were determined using a two‐tailed *t*‐test (B) and one‐way analysis of variance (ANOVA) (D).
**Figure S2:** IC50 values of Calycosin in HT29, HC116, and SW620 cells, along with statistical analyses of wound healing, migration, invasion, and apoptosis. (A) IC50 values of Calycosin in HT29, HC116, and SW620 cells. (B‐D) Statistical analyses of wound healing (B), migration (C), and invasion (D) in HT29, HC116, and SW620 cells treated with Calycosin. (E) Statistical analysis of apoptosis in HT29, HC116, and SW620 cells following Calycosin treatment. Data are presented as mean ± standard error of the mean (SEM). *p* values and significance were determined using two‐tailed *t*‐tests (B–E).
**Figure S3:** Statistical analysis of calycosin and ferrostatin‐1 on the expression of ferroptosis‐related proteins in HT29, HC116, and SW620 cells. (A) Statistical graph of the expression of ferroptosis‐related proteins GPX4, FTH1, NOX1, and ACSL4 in HT29, HC116, and SW620 cells after treatment with calycosin for 24 h. (B) Statistical analysis of the expression of ferroptosis‐related proteins in SW620 cells in the DMSO + calycosin and ferrostatin‐1 + calycosin groups. (C) Flow cytometry detection of the apoptosis proportion of SW620 cells in the DMSO + calycosin and ferrostatin‐1 + calycosin groups. Data are presented as mean ± SEM. *p* values and significance were determined by two‐tailed *t*‐tests (A–C).
**Figure S4:** Statistical analysis of apoptosis and ferroptosis‐related protein expression in HT29 and SW620 cells. (A) Statistical analysis of CYP1B1 protein expression in the control group (sh_NC) and CYP1B1 knockdown group (sh_CYP1B1). (B) Statistical analysis of the effects of CYP1B1 knockdown on apoptosis in SW620 and HT29 cells. (C) Statistical analysis of CYP1B1, GPX4, and ACSL4 protein expression in the control group (sh_NC) and CYP1B1 knockdown group (sh_CYP1B1). (D) Statistical analysis of CYP1B1 protein expression in the control group (OE_NC) and overexpression group (OE_CYP1B1). (E) Statistical analysis of the effects of CYP1B1 overexpression on apoptosis in SW620 and HT29 cells. (F) Statistical analysis of CYP1B1, GPX4, and ACSL4 protein expression in the control group (OE_NC) and CYP1B1 overexpression group (OE_CYP1B1). (G) Wound‐healing assay to evaluate the effects of calycosin on the migration ability of SW620 and HT29 cells with CYP1B1 overexpression. Scale bar = 100 μm. (H) Transwell assay to assess the effects of calycosin on the migration and invasion abilities of SW620 and HT29 cells with CYP1B1 overexpression. Scale bar = 50 μm. Data are presented as mean ± SEM. *p* values and significance were determined by two‐tailed *t*‐tests (A–F).
**Figure S5:** Molecular mechanism of CYP1B1 regulating GPX4. (A, B) Statistical analysis of the protein expression of cytoplasmic CYP1B1, nuclear phosphorylated SP1 (p‐SP1), and TFAP2A in HT29 and SW620 cells after CYP1B1 knockdown (A) and calycosin treatment (B). (C, D) Protein expression levels of cytoplasmic CYP1B1, p‐AKT1, p‐ERK1/2, and nuclear p‐SP1 in HT29 and SW620 cells after CYP1B1 knockdown (C) and calycosin treatment (D). (E) Expression levels and statistical analysis of p‐AKT, GPX4, and p‐SP1 in HT29 and SW620 cells after treatment with AKT inhibitor (MK‐2206). Data are presented as mean ± SEM. *p* values and significance were determined by two‐tailed *t*‐tests (A–E).

**Table S1:** Whole‐genome transcriptome sequencing.

**Table S2:** Differentially Expressed Genes.

**Table S3:** Genes related to cell apoptosis, necrosis, and ferroptosis.

## Data Availability

The data that support the findings of this study are available from the corresponding author upon reasonable request.
