# Supplementary material for: Calycosin Targets the CYP1B1‐AKT/SP1‐GPX4 Axis to Modulate Ferroptosis in Colorectal Carcinogenesis
Source: Phytother Res. 2026 Jan 6;40(2):721–36. doi: 10.1002/ptr.70172 (PMC12879287; doi:10.1002/ptr.70172)
Supplement: Supplementary file 1 — Figure S1: Effects of three small‐molecule compounds derived from traditional Chinese medicine on body weight, organ toxicity, and metabolic function in BALB/c‐nu mice. (A) Schematic diagram of the administration of Calycosin, (+)‐Gallocatechin and Ziyuglycoside I in BALB/c‐nu mice. (B) Line graph depicting changes in mouse body weight. (C) Gross photographs and pathological results of the heart, liver, spleen, lungs, kidneys, and brain. Histopathological examination of the heart, spleen, lungs, and kidneys was performed using hematoxylin and eosin (HE) staining, while liver tissue lesions were assessed via Oil Red O staining, and brain tissue lesions were evaluated using Nissl staining. Scale bar = 100 μm. (D) Expression levels of alanine transaminase (ALT) and aspartate aminotransferase (AST) in peripheral blood after model establishment. Data are presented as mean ± standard error of the mean (SEM). p values and significance were determined using a two‐tailed t‐test (B) and one‐way analysis of variance (ANOVA) (D). Figure S2: IC50 values of Calycosin in HT29, HC116, and SW620 cells, along with statistical analyses of wound healing, migration, invasion, and apoptosis. (A) IC50 values of Calycosin in HT29, HC116, and SW620 cells. (B‐D) Statistical analyses of wound healing (B), migration (C), and invasion (D) in HT29, HC116, and SW620 cells treated with Calycosin. (E) Statistical analysis of apoptosis in HT29, HC116, and SW620 cells following Calycosin treatment. Data are presented as mean ± standard error of the mean (SEM). p values and significance were determined using two‐tailed t‐tests (B–E). Figure S3: Statistical analysis of calycosin and ferrostatin‐1 on the expression of ferroptosis‐related proteins in HT29, HC116, and SW620 cells. (A) Statistical graph of the expression of ferroptosis‐related proteins GPX4, FTH1, NOX1, and ACSL4 in HT29, HC116, and SW620 cells after treatment with calycosin for 24 h. (B) Statistical analysis of the expression of ferroptos [file PTR-40-721-s004.docx]

**Supplementary Figures for**

Calycosin Targets the CYP1B1-AKT/SP1-GPX4 Axis to Modulate Ferroptosis in Colorectal Carcinogenesis


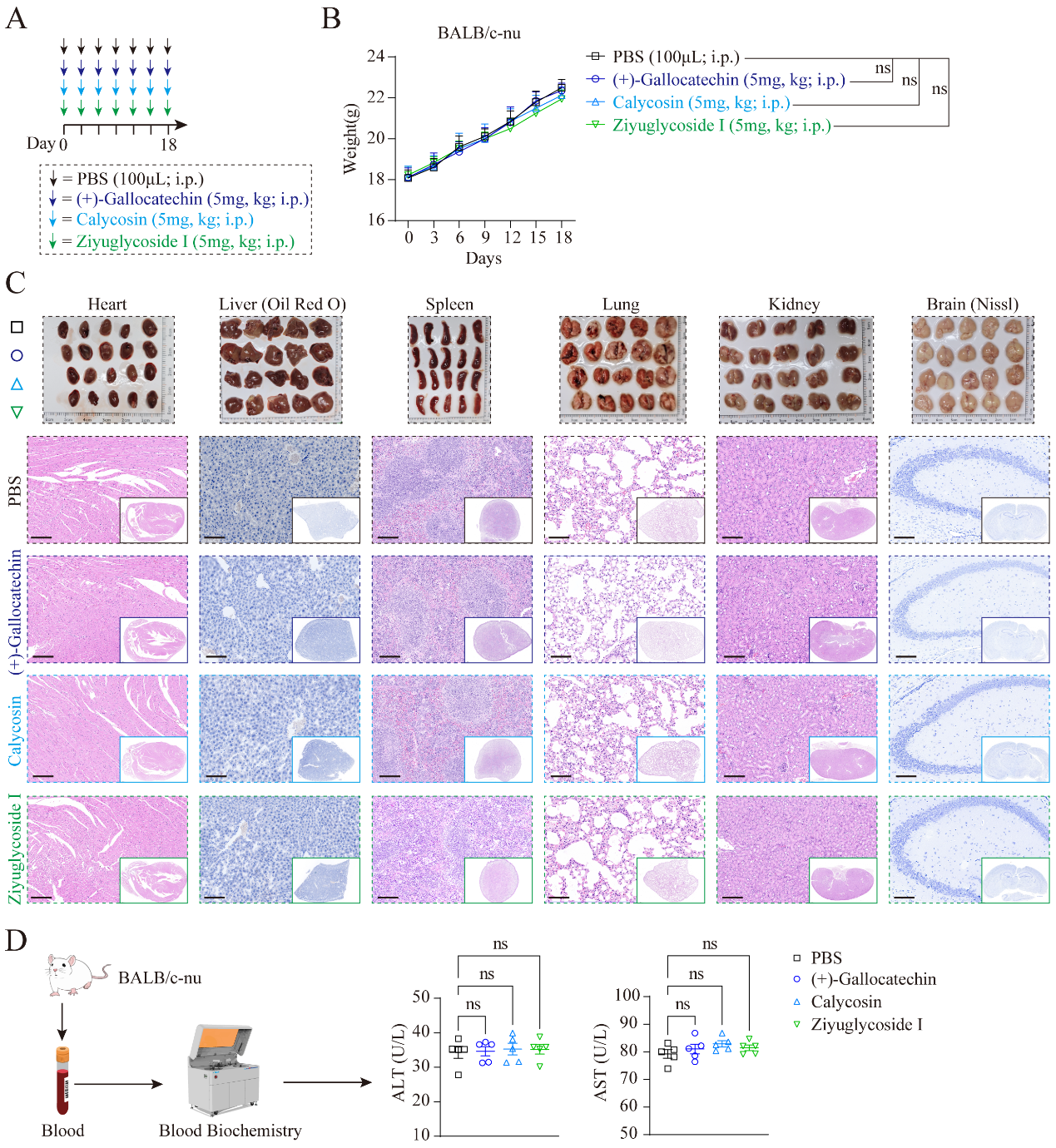


**FIGURE S1** Effects of three small-molecule compounds derived from traditional Chinese medicine on body weight, organ toxicity, and metabolic function in BALB/c-nu mice. (A) Schematic diagram of the administration of Calycosin, (+)-Gallocatechin and Ziyuglycoside I in BALB/c-nu mice. (B) Line graph depicting changes in mouse body weight. (C) Gross photographs and pathological results of the heart, liver, spleen, lungs, kidneys, and brain. Histopathological examination of the heart, spleen, lungs, and kidneys was performed using hematoxylin and eosin (HE) staining, while liver tissue lesions were assessed via Oil Red O staining, and brain tissue lesions were evaluated using Nissl staining. Scale bar = 100 μm. (D) Expression levels of alanine transaminase (ALT) and aspartate aminotransferase (AST) in peripheral blood after model establishment. Data are presented as mean ± standard error of the mean (SEM). *P*-values and significance were determined using a two-tailed t-test (B) and one-way analysis of variance (ANOVA) (D).


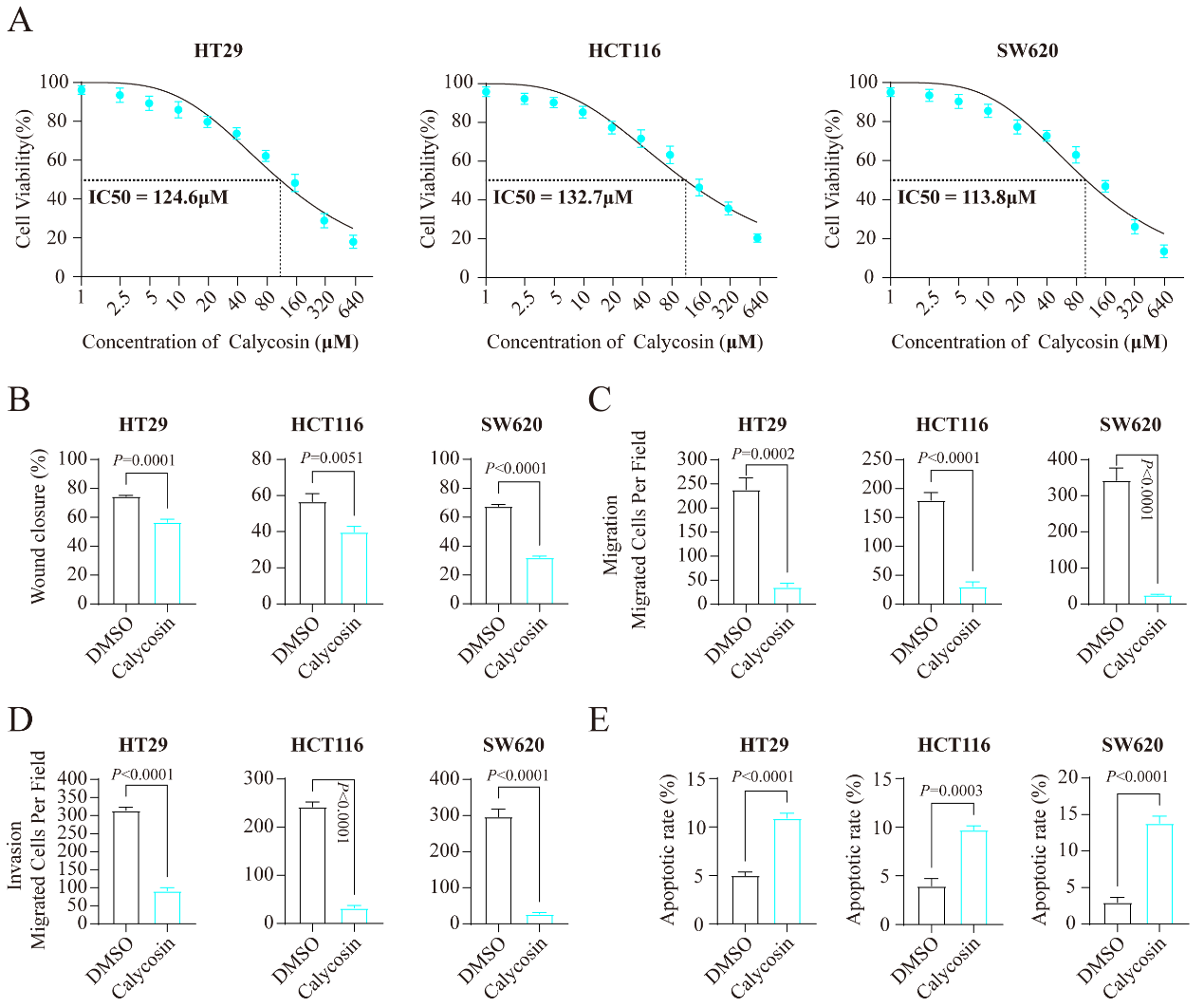


**FIGURE S2** IC50 values of Calycosin in HT29, HC116, and SW620 cells, along with statistical analyses of wound healing, migration, invasion, and apoptosis. (A) IC50 values of Calycosin in HT29, HC116, and SW620 cells. (B-D) Statistical analyses of wound healing (B), migration (C), and invasion (D) in HT29, HC116, and SW620 cells treated with Calycosin. (E) Statistical analysis of apoptosis in HT29, HC116, and SW620 cells following Calycosin treatment. Data are presented as mean ± standard error of the mean (SEM). *P*-values and significance were determined using two-tailed t-tests (B, C, D, E).


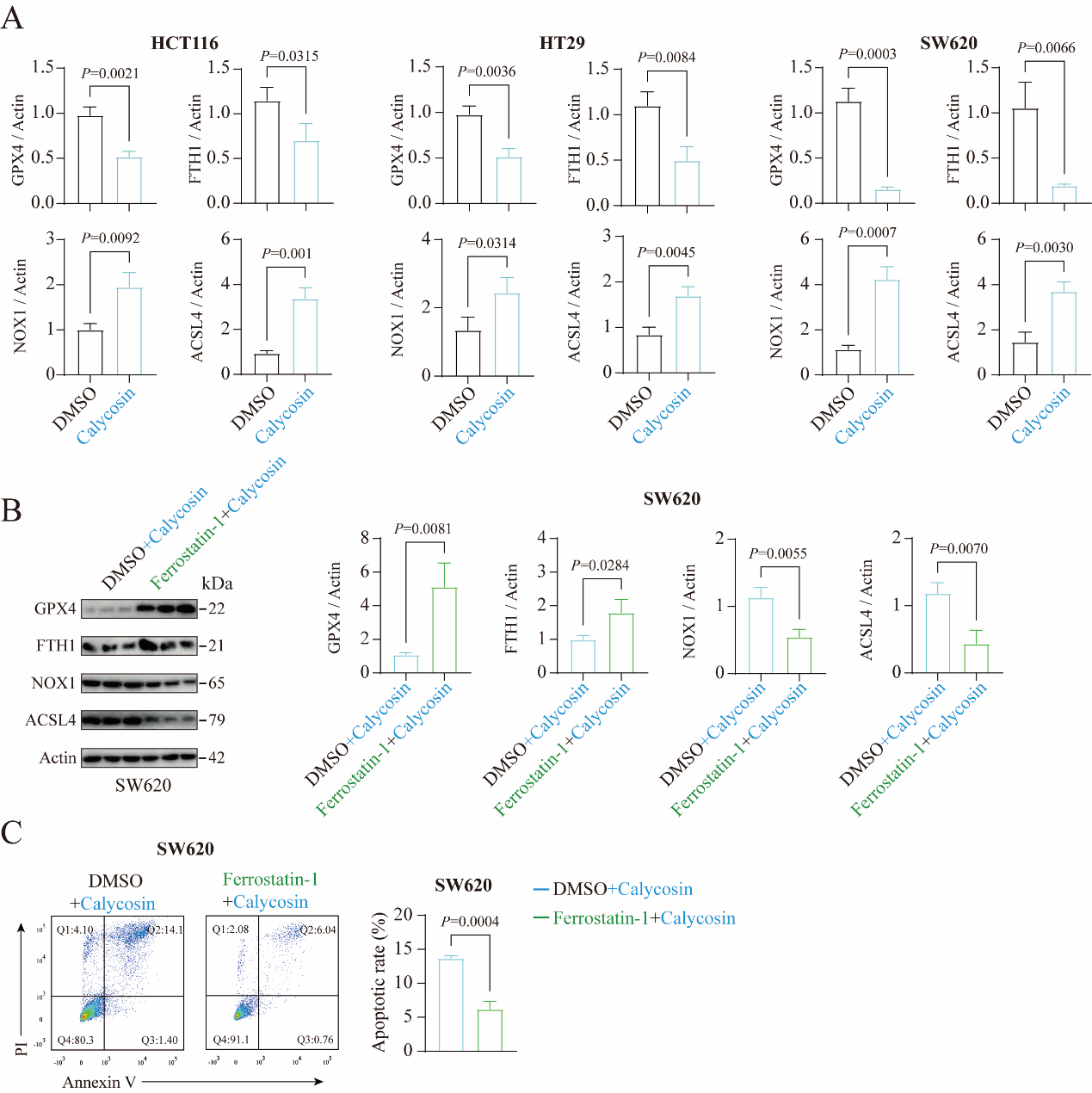


**FIGURE S3** Statistical analysis of calycosin and ferrostatin-1 on the expression of ferroptosis-related proteins in HT29, HC116, and SW620 cells. (A) Statistical graph of the expression of ferroptosis-related proteins GPX4, FTH1, NOX1, and ACSL4 in HT29, HC116, and SW620 cells after treatment with calycosin for 24 h. (B) Statistical analysis of the expression of ferroptosis-related proteins in SW620 cells in the DMSO + calycosin and ferrostatin-1 + calycosin groups. (C) Flow cytometry detection of the apoptosis proportion of SW620 cells in the DMSO + calycosin and ferrostatin-1 + calycosin groups. Data are presented as mean ± SEM. P-values and significance were determined by two-tailed t -tests (A, B, C).


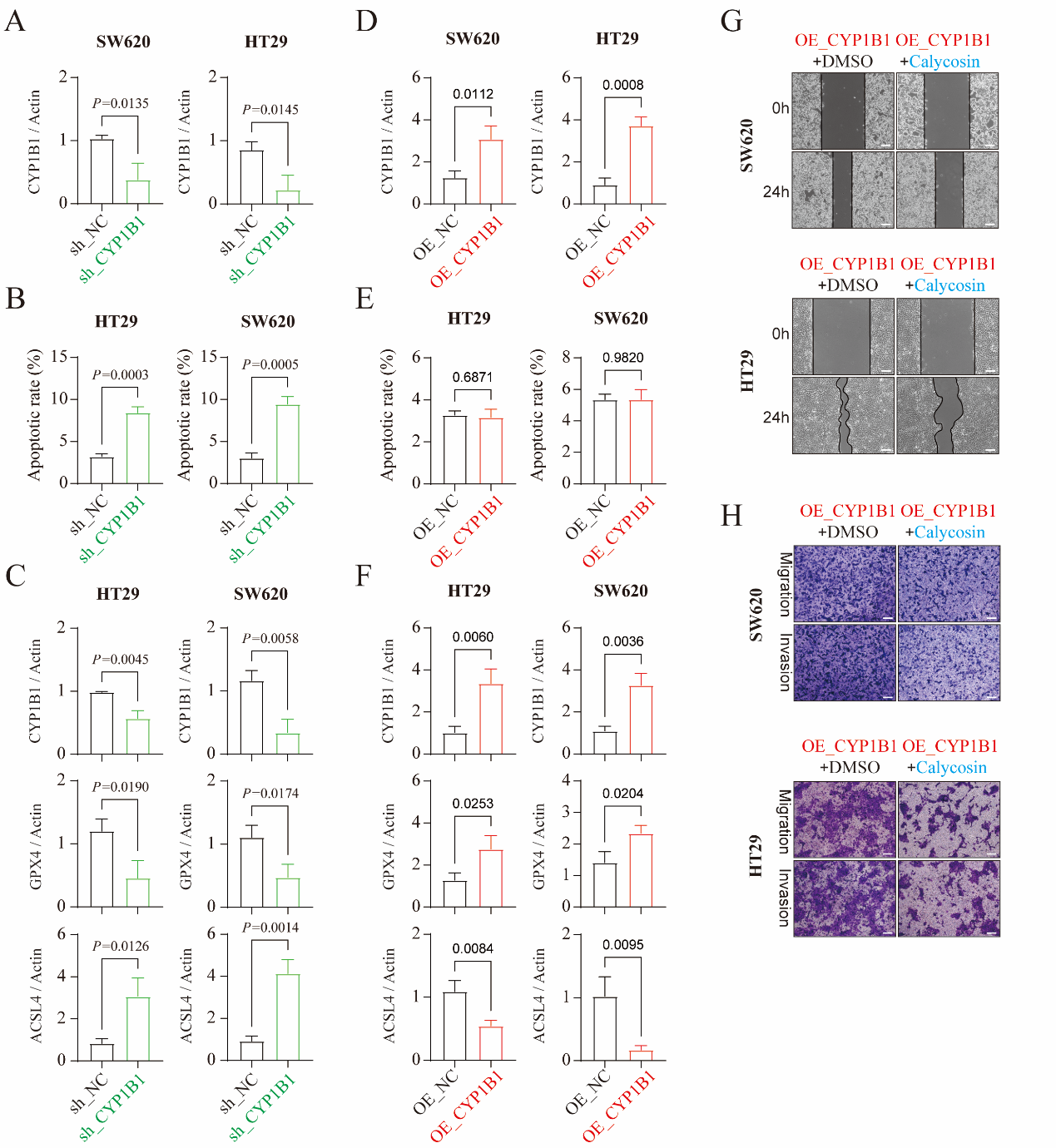


**FIGURE S4** Statistical analysis of apoptosis and ferroptosis - related protein expression in HT29 and SW620 cells. (A) Statistical analysis of CYP1B1 protein expression in the control group (sh_NC) and CYP1B1 knockdown group (sh_CYP1B1). (B) Statistical analysis of the effects of CYP1B1 knockdown on apoptosis in SW620 and HT29 cells. (C) Statistical analysis of CYP1B1, GPX4, and ACSL4 protein expression in the control group (sh_NC) and CYP1B1 knockdown group (sh_CYP1B1). (D) Statistical analysis of CYP1B1 protein expression in the control group (OE_NC) and overexpression group (OE_CYP1B1). (E) Statistical analysis of the effects of CYP1B1 overexpression on apoptosis in SW620 and HT29 cells. (F) Statistical analysis of CYP1B1, GPX4, and ACSL4 protein expression in the control group (OE_NC) and CYP1B1 overexpression group (OE_CYP1B1). (G) Wound - healing assay to evaluate the effects of calycosin on the migration ability of SW620 and HT29 cells with CYP1B1 overexpression. Scale bar = 100μm. (H) Transwell assay to assess the effects of calycosin on the migration and invasion abilities of SW620 and HT29 cells with CYP1B1 overexpression. Scale bar = 50μm. Data are presented as mean ± SEM. P - values and significance were determined by two - tailed t - tests (A, B, C, D, E, F).


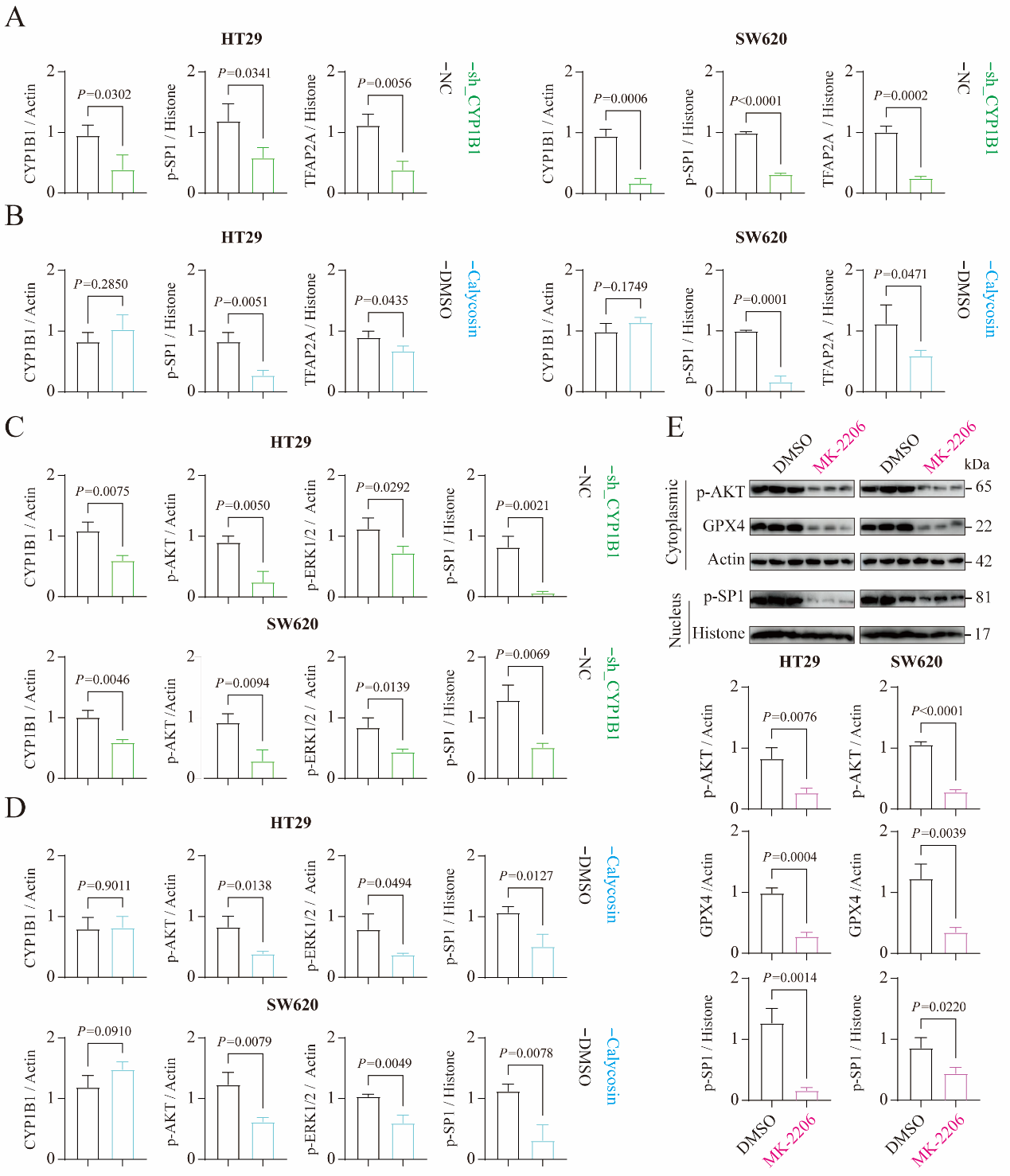


**FIGURE S5** Molecular mechanism of CYP1B1 regulating GPX4. (A, B) Statistical analysis of the protein expression of cytoplasmic CYP1B1, nuclear phosphorylated SP1 (p - SP1), and TFAP2A in HT29 and SW620 cells after CYP1B1 knockdown (A) and calycosin treatment (B). (C, D) Protein expression levels of cytoplasmic CYP1B1, p - AKT1, p - ERK1/2, and nuclear p - SP1 in HT29 and SW620 cells after CYP1B1 knockdown (C) and calycosin treatment (D). (E) Expression levels and statistical analysis of p - AKT, GPX4, and p - SP1 in HT29 and SW620 cells after treatment with AKT inhibitor (MK - 2206). Data are presented as mean ± SEM. P - values and significance were determined by two - tailed t - tests (A, B, C, D, E).
